# Supplementary material for: TASOR epigenetic repressor cooperates with a CNOT1 RNA degradation pathway to repress HIV
Source: Nat Commun. 2022 Jan 10;13:66. doi: 10.1038/s41467-021-27650-5 (PMC8748822; doi:10.1038/s41467-021-27650-5)
Supplement: Supplementary file 1 — Supplementary Information [file 41467_2021_27650_MOESM1_ESM.pdf]

Supplementary Table 1

Domain fold recognition and eukaryotic function predictions of TASOR 1-900

**TASOR 1-900 aa**	**pGenTHREADER, Fold Recognition**		
Confidence	Target protein (Human unless specified)	Net Score	p-value
CERT	PARP12	68.105	7,00E-06
CERT	PARP14	67.410	8,00E-06
CERT	PARP15	61.019	3,00E-05
CERT	PARP13	58.639	6,00E-05
HIGH	PARP1	52.145	3,00E-04
HIGH	PARP10	47.367	8,00E-04
MEDIUM	RCD1 (*Arabidopsis thaliana*)	45.574	1,00E-03
MEDIUM	RNA Helicase Aquarius	44.130	2,00E-03
MEDIUM	ADRM1	43.744	2,00E-03
MEDIUM	Spoc domain of FPA (*Arabidopsis thaliana*)	43.610	2,00E-03
MEDIUM	Ovotransferrin (*Anas platyrhynchos*)	43.475	2,00E-03
MEDIUM	Anthrax Edema Factor	43.414	2,00E-03
MEDIUM	Ovotransferrin (*Gallus gallus*)	40.177	4,00E-03
MEDIUM	Ku	39.673	5,00E-03
MEDIUM	C-Src	39.475	5,00E-03
	**Biological Process Predictions**		
GO term	Biological process	Prob	SVM reliability
GO:0008380	RNA splicing	0.937	High
GO:0019222	regulation of metabolic process	0.918	High
GO:0000398	mRNA splicing, *via* spliceosome	0.891	High
GO:0000375	RNA splicing, *via* transesterification reactions	0.868	High
GO:0034645	cellular macromolecule biosynthetic process	0.832	High
GO:0006396	RNA processing	0.813	High
GO:0006351	transcription, DNA-templated	0.813	High
GO:2001141	regulation of RNA biosynthetic process	0.749	High
GO:1903506	regulation of nucleic acid-templated transcription	0.740	High
GO:0006397	mRNA processing	0.734	High
GO:0010468	regulation of gene expression	0.676	High
GO:0051171	regulation of nitrogen compound metabolic process	0.633	High
GO:0006810	transport	0.614	High
GO:0010629	negative regulation of gene expression	0.578	High
GO:0009059	macromolecule biosynthetic process	0.568	High
GO:0051252	regulation of RNA metabolic process	0.527	High
GO:0006355	regulation of transcription, DNA-templated	0.517	High

Supplementary Table 1: Domain fold recognition and eukaryotic function predictions of TASOR 1-900.

The first 900 amino-acid sequence of TASOR (NCBI Reference Sequence: NP_001106207.1) was loaded on the PSIPRED server (http://bioinf.cs.ucl.ac.uk/psipred/) for Domain fold recognition (pGenTHREADER) and Eukaryotic function predictions (FFPred3). Results were sorted according to confidence/reliability. Indicated p-values were calculated by pGenTHREADER (Lobley et al., 2009).

Supplementary Table 2

Primer sequences that were used for qPCR analyses

Primer name	Sequence 5'-3'
F-Firefly Luc	CTCACTGAGACTACATCAGC
R-Firefly Luc	TCCAGATCCACAACCTTCG
F-MORC2	ACATGAAGACGCAGGAAGAG
R-MORC2	ACTTCCAAGGGCAATTTCTT
F-TNFα	CTCTTCTGCCTGCTGCACTTTG
R-TNFα	ATGGGCTACAGGCTTGTCACTC
F-TUG1	TAATTGCCCAGCATCCGTTCCA
R-TUG1	CATGTTCACCCACAAAGCTCAAGG
F-18S rRNA	GTAACCCGTTGAACCCCATT
R-18S rRNA	CCATCCAATCGGTAGTAGCG
F-HIV-1 Nuc0	ATCTACCACACACAAGGCTAC
R-HIV-1 Nuc0	GTACTAACTTGAAGCACCATCC
F-HIV-1 Nuc1	AGTAGTGTGTGCCCGTCTGT
R-HIV-1 Nuc1	TTGGCGTACTCACCAGTCGC
F-Chr12.Neg Ctrl	ATGGTTGCCACTGGGGATCT
R-Chr12.Neg Ctrl	TGCCAAAGCCTAGGGGAAGA
F-GADPH	ATGGGGAAGGTGAAGGTCG
R-GAPDH	AGTTAAAAGCAGCCCTGGTG
F-HIV-1 US RNA	GTGTGGAAAATCTCTAGCAGTGG
R-HIV-1 US RNA	CGCTCTCGCACCCATCTC

Supplementary Table 2: List of primers used in this study for qPCRs.

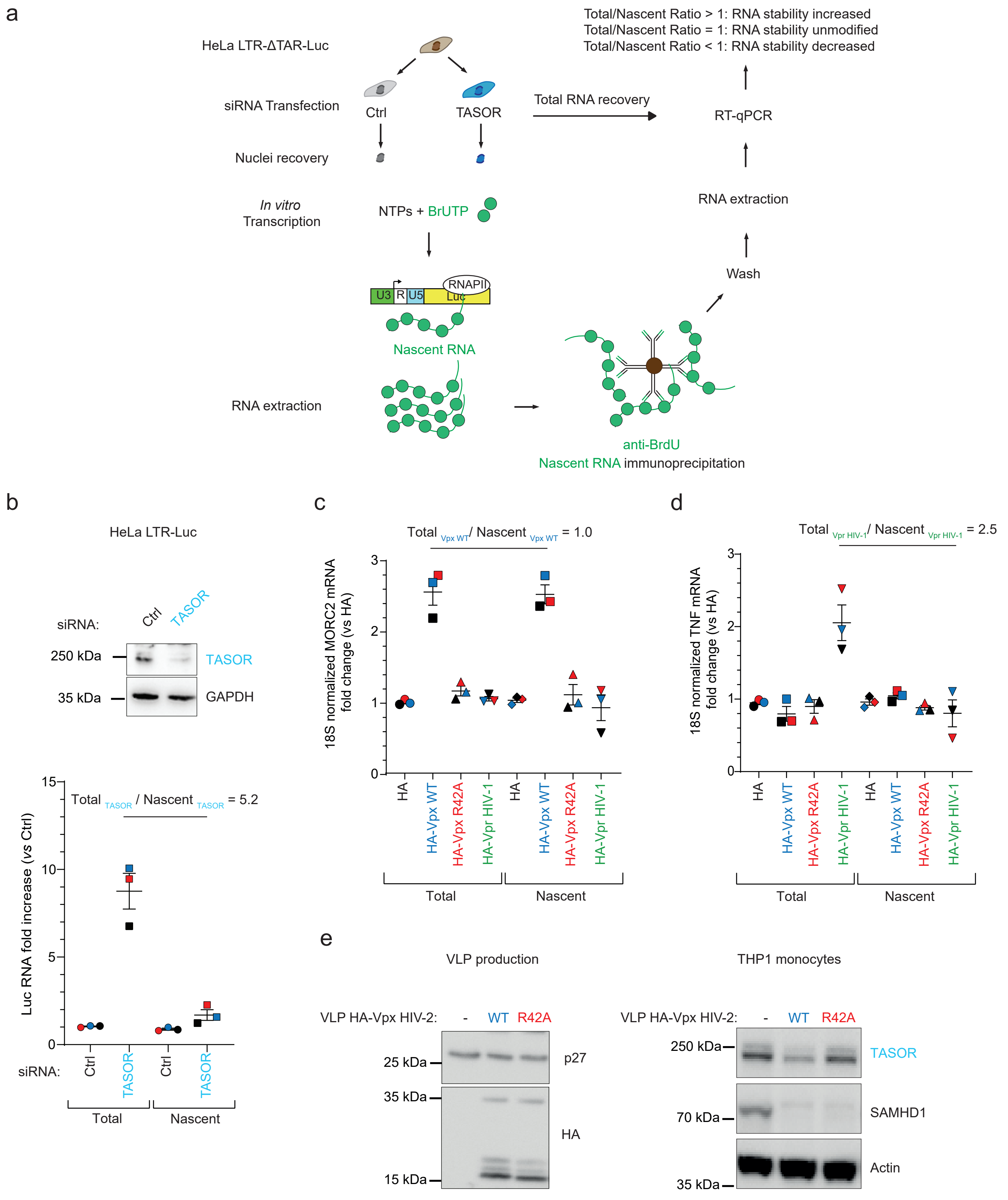
a
HeLa LTR-ΔTAR-Luc
siRNA Transfection
Ctrl
TASOR
Nuclei recovery
In vitro Transcription
NTPs + BrUTP
U3 R U5 Luc
RNAPII
Nascent RNA
RNA extraction
anti-BrdU
Nascent RNA immunoprecipitation
Wash
RNA extraction
RT-qPCR
Total RNA recovery
Total/Nascent Ratio > 1: RNA stability increased
Total/Nascent Ratio = 1: RNA stability unmodified
Total/Nascent Ratio < 1: RNA stability decreased
b
HeLa LTR-Luc
siRNA:
Ctrl
TASOR
250 kDa
35 kDa
TASOR
GAPDH
Luc RNA fold increase (vs Ctrl)
Total TASOR / Nascent TASOR = 5.2
15
10
5
0
Total
Nascent
c
Total Vpx WT / Nascent Vpx WT = 1.0
18S normalized MORC2 mRNA fold change (vs HA)
3
2
1
0
HA
HA-Vpx WT
HA-Vpx R42A
HA-Vpr HIV-1
Total
Nascent
d
Total Vpr HIV-1 / Nascent Vpr HIV-1 = 2.5
18S normalized TNF mRNA fold change (vs HA)
e
VLP production
VLP HA-Vpx HIV-2:
-
WT
R42A
25 kDa
35 kDa
15 kDa
p27
HA
THP1 monocytes
250 kDa
70 kDa
35 kDa
TASOR
SAMHD1
Actin

Figure S1

Figure S1:

a *Nuclear Run On* strategy employed to characterize TASOR's function in the mRNA metabolism pathway HeLa HIV-1 LTRΔTAR-Luc cells. **b** TASOR negatively impacts LTR-driven Luc transcript at a post-transcriptional step. *Nuclear Run On* performed in HeLa HIV-1 LTR-Luc after 72h of siCtrl or siTASOR transfection. (n=3; each color represents one different independent experiment, mean and SEM are showed). **c-d** HIV-2 Vpx mimics siRNA-mediated TASOR silencing. HeLa HIV-1 LTRΔTAR-Luc were transfected with pAS1B-HA, pAS1B-HA-Vpx WT HIV-2, or pAS1B-HA-Vpx R42A HIV-2, or pAS1B-HA-Vpr HIV-1 for 48h. *Nuclear Run On* experiments were undertaken to measure MORC2 (**c**), TNFα (**d**) RNA levels at the transcriptional or post-transcriptional steps (For panels **c** and **d** n=3 with each color representing one different independent experiment. The mean and SEM are showed). **e** Vpx R42A mutant is unable to trigger TASOR degradation while being as efficient as Vpx WT in inducing the degradation of SAMHD1. Vpx WT and R42A equivalently incorporated into VLPs were delivered into the THP1 cell line for 24h (n>3). Source data are provided as a Source Data file

a

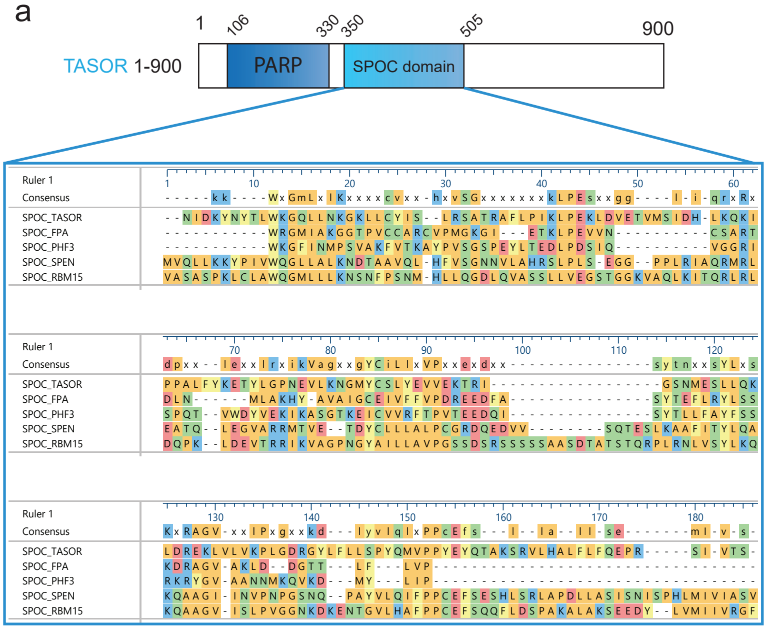

b

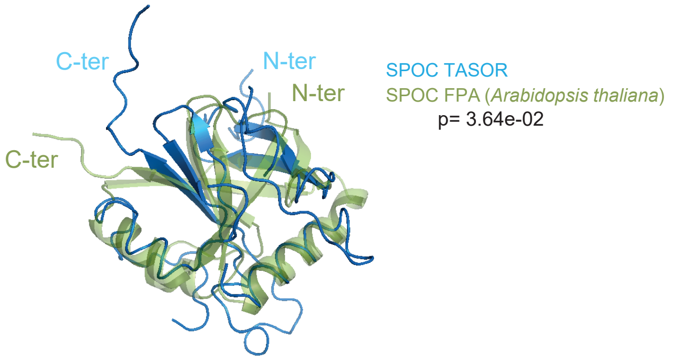

c

Bait: TASOR 1-900

Prey: Human Macrophage Proteome

Score	TASOR 1-900 partner	Partner peptide
A	CYFIP1	24-413
A	GCC2	412-551
A	LXRα	84-328
B	Actinin-1	258-586
B	IMMT	469-728
B	TGFBRAP1	399-816
C	ARID1A	1880-2134
C	CNOT1	1317-1499
C	MPP8	383-831
D	DHX29	285-748
D	DHX30	138-485
D	EIF4G1	1173-1400
D	EP300	1469-1699
D	KDM5C	753-1108
D	MATR3	92-427
D	SAMHD1	1-255
D	SUPT6H	240-695
D	ZFC3H1	1581-1685

d

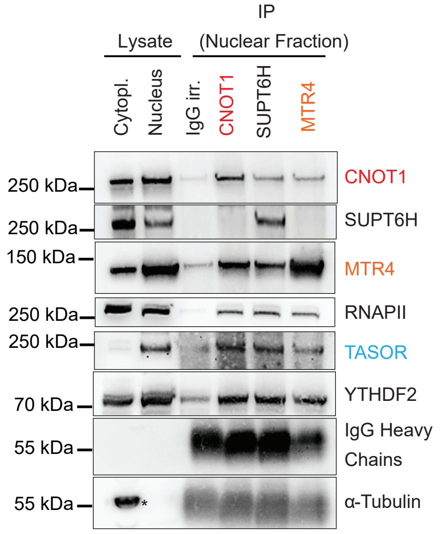

e

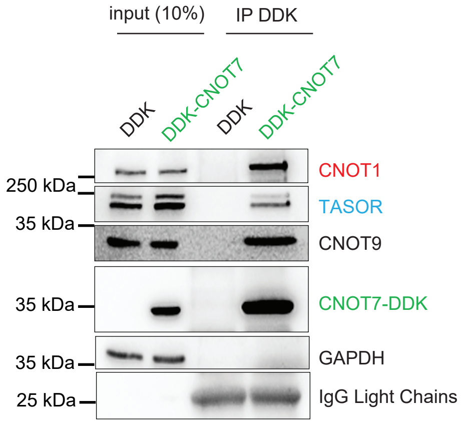

f

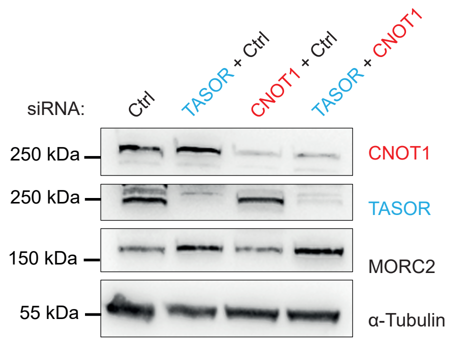

g

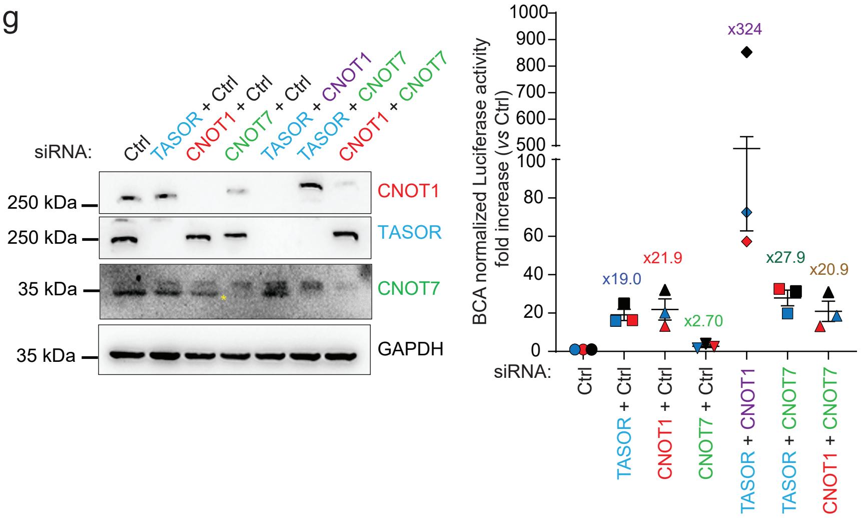

Figure S2

Figure S2:

a Structure prediction of TASOR first 900 amino-acids detected a PARP domain and a SPOC domain. SPOC domains from different SPOC containing proteins were aligned using clustal Omega. Proteins and their identifier: *Homo sapiens* TASOR isoform1: Q9UK61-1; *Arabidopsis thaliana* FPA isoform1: Q8LPQ9-1; *Homo sapiens* PHF3 isoform1: Q92576-1; Homo sapiens SPEN: Q96T58-1; *Homo sapiens* RBM15 isoform1: Q96T37-1. **b** Overlay of TASOR SPOC domain predicted structure (RaptorX) with the *Arabidopsis thaliana* FPA SPOC domain (PDB:5KXF [http://doi.org/10.2210/pdb5KXF/pdb]). **c** A yeast two-hybrid (Y-2-H) screen was performed using a human macrophage cDNA library to identify interacting proteins with TASOR (1-900). Interacting partners were assigned a predicted biological score from A-F to assess the confidence of an interaction being specific (with A indicating very high confidence, and F indicating experimentally determined artifacts). TASOR's interaction site (aa) on the identified partner is shown in the 'partner peptide' column. **d** Endogenous TASOR interacts with the histone chaperone and transcription elongation factor SUPT6H as well as endogenous CNOT1 and MTR4. HeLa HIV-1 LTRΔTAR-Luc cells were fractionated and CNOT1, SUPT6H, MTR4 immunoprecipitations were performed from nuclear extracts (n>3 between TASOR and CNOT1, MTR4 and n=3 with SUPT6H). **e** TASOR interacts with the CCR4-NOT complex deadenylase CNOT7. DDK and TASOR-DDK vectors were transfected in HeLa cells and anti-DDK immunoprecipitation was performed. GAPDH is a negative control (n=3 between TASOR and CNOT7). **f** Western-blot associated with the *Nuclear Run On* data presented on Fig.2 (n=3) **g** Cooperation between the CCR4-NOT complex deadenylase CNOT7 and TASOR in the repression of HIV-1 LTR-Luc expression. Following 72h of siRNA transfections in HeLa HIV-1 LTR-ΔTAR-Luc, cells were lysed, siRNA-mediated silencing of proteins was controlled by Western-Blot and Luciferase activity was measured and normalized

on protein concentration. (n=3; each color represents one different independent experiment, mean and SEM are showed). Source data are provided as a Source Data file.

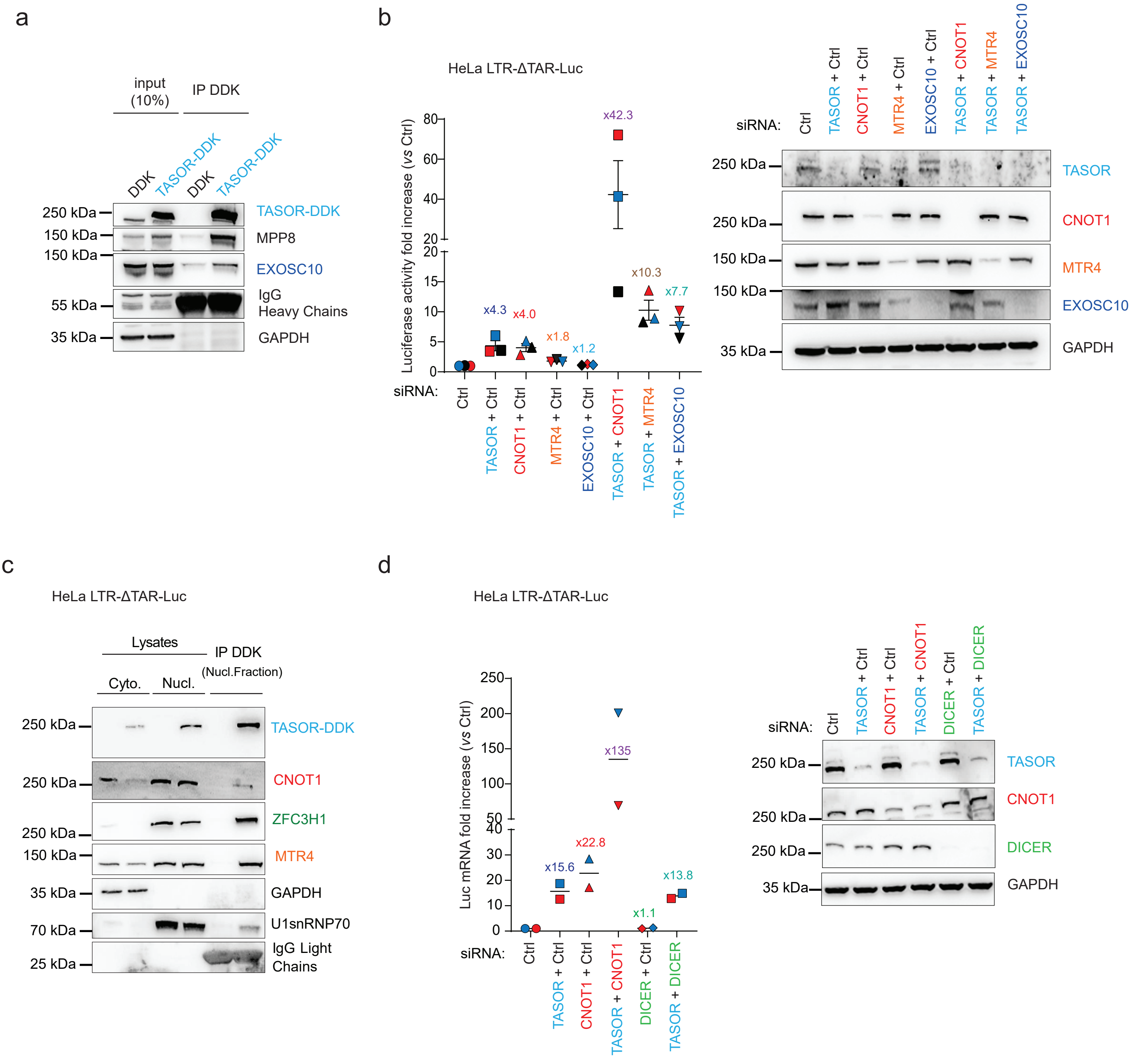
a
input (10%)
IP DDK
DDK
TASOR-DDK
DDK
TASOR-DDK
250 kDa
150 kDa
150 kDa
55 kDa
35 kDa
TASOR-DDK
MPP8
EXOSC10
IgG Heavy Chains
GAPDH
b
HeLa LTR-ΔTAR-Luc
Luciferase activity fold increase (vs Ctrl)
x42.3
x10.3
x7.7
x4.3
x4.0
x1.8
x1.2
siRNA:
Ctrl
TASOR + Ctrl
CNOT1 + Ctrl
MTR4 + Ctrl
EXOSC10 + Ctrl
TASOR + CNOT1
TASOR + MTR4
TASOR + EXOSC10
250 kDa
250 kDa
150 kDa
150 kDa
35 kDa
TASOR
CNOT1
MTR4
EXOSC10
GAPDH
c
HeLa LTR-ΔTAR-Luc
Lysates
IP DDK
(Nucl.Fraction)
Cyto.
Nucl.
250 kDa
250 kDa
250 kDa
150 kDa
35 kDa
70 kDa
25 kDa
TASOR-DDK
CNOT1
ZFC3H1
MTR4
GAPDH
U1snRNP70
IgG Light Chains
d
HeLa LTR-ΔTAR-Luc
Luc mRNA fold increase (vs Ctrl)
x135
x22.8
x15.6
x13.8
x1.1
siRNA:
Ctrl
TASOR + Ctrl
CNOT1 + Ctrl
TASOR + CNOT1
DICER + Ctrl
TASOR + DICER
250 kDa
250 kDa
250 kDa
35 kDa
TASOR
CNOT1
DICER
GAPDH

Figure S3

Figure S3:

a TASOR interacts with the exosome factor EXOSC10. HeLa HIV-1 LTRΔTAR-Luc cells were transfected with DDK or TASOR-DDK constructs for 48h and an-anti DDK immunoprecipitation was performed (n>3) **b** TASOR cooperates with TRAMP-like/NEXT/PAXT components MTR4 and the nuclear RNA exosome factor EXOSC10 in the repression of HIV-1 LTR-Luc expression. After 72h of siRNA transfections in HeLa HIV-1 LTR-ΔTAR-Luc, cells were lysed, siRNA-mediated silencing of proteins was controlled by Western-Blot and Luciferase activity was measured and normalized on protein concentration. (n=3; each color represents one different independent experiment, mean and SEM are showed). **c** TASOR interacts with the nuclear TRAMP-like/PAXT complex member ZFC3H1. HeLa HIV-1 LTRΔTAR-Luc cells transfected with DDK or TASOR-DDK constructs were fractionated to recover the nuclear fraction. The endogenous PAXT complex components ZFC3H1 and MTR4 were revealed associated with TASOR-DDK along with CNOT1 and the 5'splice donor site recognizing factor U1SNRP70. GAPDH is a negative control (n>3 between TASOR and ZFC3H1, MTR4, CNOT1, n=2 between TASOR and U1SNRP70). **d** RNAi-pathway factor DICER has no role and does not cooperate with TASOR in the repression of the HIV-1 LTR-Luc expression. After 72h of siRNA transfections in HeLa HIV-1 LTR-ΔTAR-Luc, cells were lysed, siRNA-mediated silencing of proteins was controlled by Western-Blot and Luciferase activity was measured and normalized on protein concentration (n=2; each color represents one different independent experiment). Source data are provided as a Source Data file.

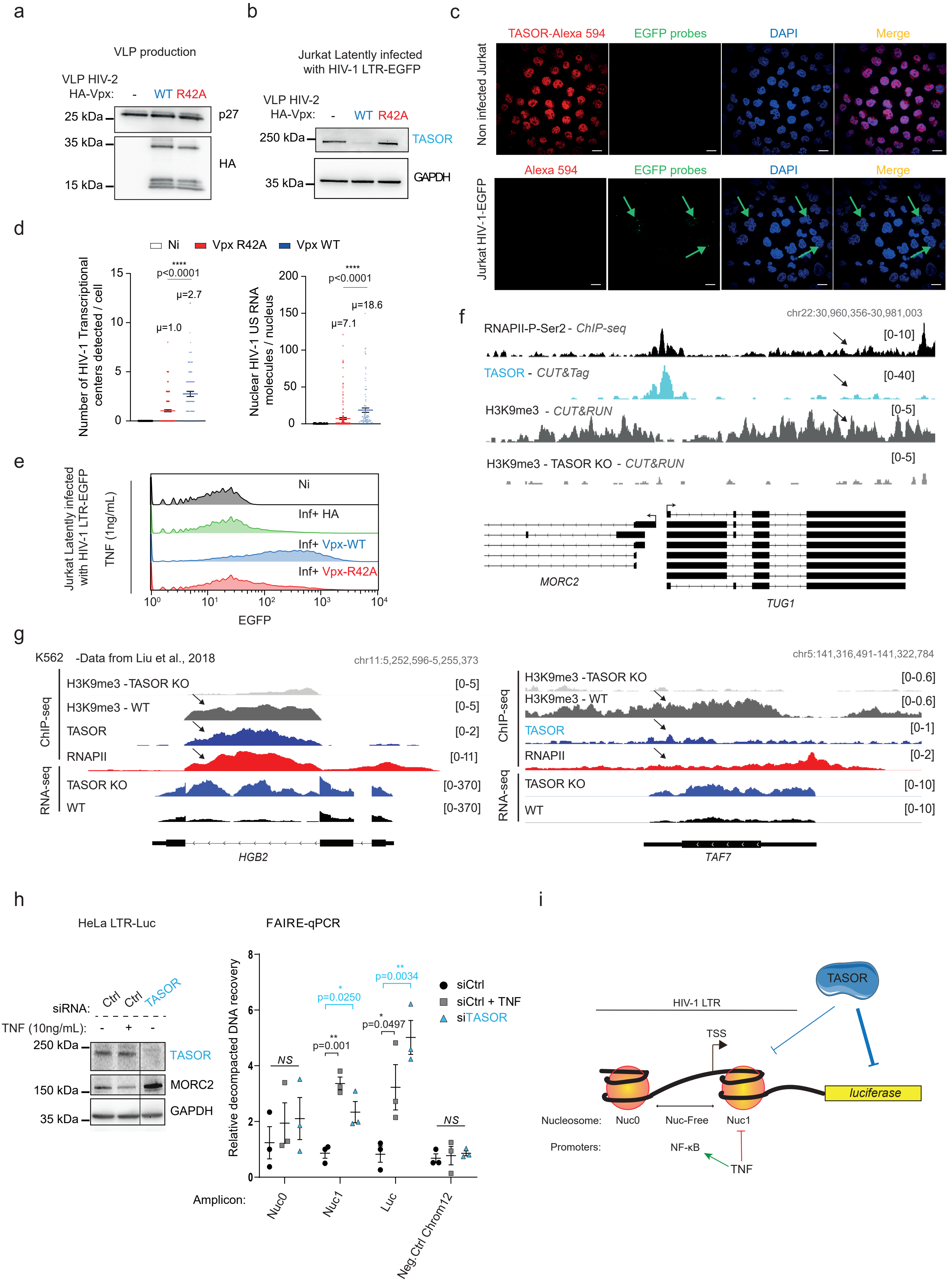
a
VLP production
VLP HIV-2 HA-Vpx: - WT R42A
25 kDa
35 kDa
15 kDa
p27
HA
b
Jurkat Latently infected with HIV-1 LTR-EGFP
VLP HIV-2 HA-Vpx: - WT R42A
250 kDa
35 kDa
TASOR
GAPDH
c
TASOR-Alexa 594
EGFP probes
DAPI
Merge
Non infected Jurkat
Alexa 594
Jurkat HIV-1-EGFP
d
Ni
Vpx R42A
Vpx WT
Number of HIV-1 Transcriptional centers detected / cell
p<0.0001
μ=2.7
μ=1.0
Nuclear HIV-1 US RNA molecules / nucleus
p<0.0001
μ=18.6
μ=7.1
e
Jurkat Latently infected with HIV-1 LTR-EGFP
TNF (1ng/mL)
Ni
Inf+ HA
Inf+ Vpx-WT
Inf+ Vpx-R42A
EGFP
f
chr22:30,960,356-30,981,003
RNAPII-P-Ser2 - ChIP-seq
[0-10]
TASOR - CUT&Tag
[0-40]
H3K9me3 - CUT&RUN
[0-5]
H3K9me3 - TASOR KO - CUT&RUN
[0-5]
MORC2
TUG1
g
K562 -Data from Liu et al., 2018
chr11:5,252,596-5,255,373
ChIP-seq
H3K9me3 -TASOR KO [0-5]
H3K9me3 - WT [0-5]
TASOR [0-2]
RNAPII [0-11]
RNA-seq
TASOR KO [0-370]
WT [0-370]
HGB2
chr5:141,316,491-141,322,784
H3K9me3 -TASOR KO [0-0.6]
H3K9me3 - WT [0-0.6]
TASOR [0-1]
RNAPII [0-2]
TASOR KO [0-10]
WT [0-10]
TAF7
h
HeLa LTR-Luc
siRNA: Ctrl Ctrl TASOR
TNF (10ng/mL): - + -
250 kDa
150 kDa
35 kDa
TASOR
MORC2
GAPDH
FAIRE-qPCR
Relative decompacted DNA recovery
siCtrl
siCtrl + TNF
siTASOR
NS
p=0.001
p=0.0250
p=0.0497
p=0.0034
NS
Amplicon:
Nuc0
Nuc1
Luc
Neg Ctrl Chrom12
i
TASOR
HIV-1 LTR
TSS
luciferase
Nucleosome: Nuc0 Nuc-Free Nuc1
Promoters: NF-κB TNF

Figure S4

Figure S4:

a Vpx WT and R42A incorporated into VLPs were delivered into the latently HIV-1 LTR-EGFP infected Jurkat cell line (n>3). **b** TASOR protein expression was assessed by Western-Blot (n>3). **c** Labelling with TASOR-Alexa 594 and secondary antibody only was performed to verify the absence of overlapping signals with EGFP probe signal. Green arrows indicate detection of the HIV-1 transcripts. Scale bar: 10µM (number of observed cells >30) **d** TASOR depletion increases the transcription of HIV-1 LTR-EGFP provirus and the quantity of nuclear HIV-1 unspliced RNAs in infected Jurkat cells. n= 206 nuclei and n= 97 nuclei for the Vpx R42A and WT conditions respectively. µ represents the mean, SEM is shown. two-sided unpaired t-test was applied. **e** These cells were analyzed by flow cytometry to evaluate the expression of the EGFP protein and smRNA FISH experiments were performed on these samples**.** **f** Colocalization between the elongating RNAPII and HUSH-dependent H3K9me3 deposition on the *TUG1* gene in HeLa cells. RNAPII-P-Ser2 ChiP-seq data ENCFF738UXP are from the ENCODE project [https://www.encodeproject.org/], Michael Snyder, Stanford. The CUT&Tag and CUT&RUN data of TASOR GSM4710610 and H3K9me3 GSM4710590, GSM4710594 respectively were published by Douse et al. [12] and deposited at GEO under the accession number GSE155693 [https://www.ncbi.nlm.nih.gov/geo/query/acc.cgi?acc=GSE155693]. Black arrow shows the colocalization signals between HUSH-dependent H3K9me3 marks, TASOR and RNAPII-P-Ser2. **g** TASOR colocalizes with HUSH-dependent H3K9me3 deposition and RNAPII on the intron of *HBG2* gene in human chronic myeloid leukemia K562 cells. ChIPseq, RNAseq performed by Liu et al., [53] and Deposited at GEO under the accession number GSE95374 [https://www.ncbi.nlm.nih.gov/geo/query/acc.cgi?acc=GSE95374]. Black arrow shows the colocalization between HUSH-dependent H3K9me3 marks, TASOR and RNAPII. TASOR colocalizes with HUSH-dependent H3K9me3 deposition and RNAPII on the *TAF7* gene body

in K562 cells. **h** TASOR silencing triggers the decompaction of the coding sequence from the HIV-1 LTR Transcription Start Site (TSS). HeLa LTR-Luc cells were transfected with siCtrl or siTASOR for 72h. TNFα (10ng/mL- 4h) treatment increases transcription from the TSS and then favors Nuc1 eviction. FAIRE-qPCR was performed (n=3), SEM is shown and two-sided unpaired t-test was applied. **i** Schematic representation of HIV-1 LTR-Luc regions compacted by TASOR. Source data are provided as a Source Data file.

a

b

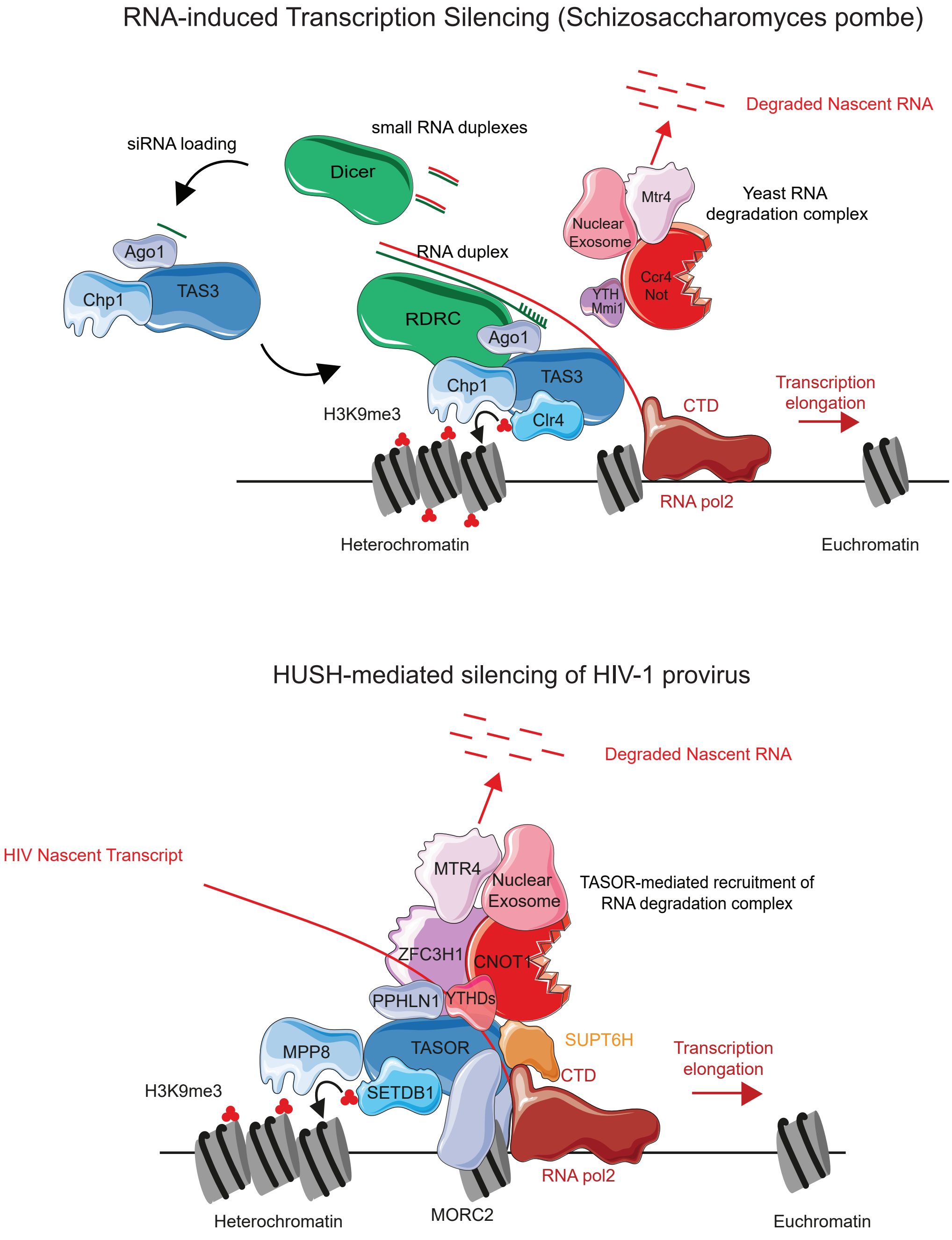

RNA-induced Transcription Silencing (Schizosaccharomyces pombe)
Degraded Nascent RNA
small RNA duplexes
siRNA loading
Dicer
Mtr4
Nuclear Exosome
Yeast RNA degradation complex
Ago1
Chp1
TAS3
RNA duplex
Ccr4 Not
YTH Mmi1
RDRC
Ago1
Chp1
TAS3
Clr4
H3K9me3
CTD
Transcription elongation
RNA pol2
Heterochromatin
Euchromatin
HUSH-mediated silencing of HIV-1 provirus
Degraded Nascent RNA
HIV Nascent Transcript
MTR4
Nuclear Exosome
TASOR-mediated recruitment of RNA degradation complex
ZFC3H1
CNOT1
PPHLN1
YTHDs
MPP8
TASOR
SUPT6H
Transcription elongation
H3K9me3
SETDB1
CTD
RNA pol2
Heterochromatin
MORC2
Euchromatin

Figure S5

Figure S5: Overall conserved model of retroelement repression by Fission Yeast RITS complex and Human HUSH complex.

a Retroelement silencing in *Schizosaccharomyces pombe*: Chp1, Tas3, Ago1 build the RITS complex which, with the help of the Histone methyl transferase Clr4, mediates retroelement silencing by spreading the H3K9me3 marks in an RNAi-dependent pathway (RDRC/DICER/siRNA) and associates with Mmi1, Mtr4, nuclear exosome in an RNAi-independent silencing pathway. **b** Integrated HIV-1 LTR provirus silencing in *Homo sapiens*: The HUSH complex interacts and follows the slowly elongating RNAPII, and recruits RNA degradation factors such as CNOT1 and members of the PAXT complex (MTR4, ZFC3H1), the nuclear RNA exosome, to eventually degrade the LTR-driven Luc RNA (this study). With the help of SETDB1, HUSH also spreads the H3K9me3 marks on the HIV-1 coding sequence as already shown in [5]. These illustrations were created using freely available Servier medical art tool at smart.servier.com.
