# Supplementary material for: TASOR epigenetic repressor cooperates with a CNOT1 RNA degradation pathway to repress HIV
Source: Nat Commun. 2022 Jan 10;13:66. doi: 10.1038/s41467-021-27650-5 (PMC8748822; doi:10.1038/s41467-021-27650-5)
Supplement: Supplementary file 3 — Reporting Summary [file 41467_2021_27650_MOESM3_ESM.pdf]

nature portfolio

Corresponding author(s): Florence Margottin-Goguet, Roy MATKOVIC

Last updated by author(s): Nov 16, 2021

# Reporting Summary

Nature Portfolio wishes to improve the reproducibility of the work that we publish. This form provides structure for consistency and transparency in reporting. For further information on Nature Portfolio policies, see our Editorial Policies and the Editorial Policy Checklist.

## Statistics

For all statistical analyses, confirm that the following items are present in the figure legend, table legend, main text, or Methods section.

| n/a | Confirmed | |
|---|---|---|
| ☐ | ☒ | The exact sample size (*n*) for each experimental group/condition, given as a discrete number and unit of measurement |
| ☐ | ☒ | A statement on whether measurements were taken from distinct samples or whether the same sample was measured repeatedly |
| ☐ | ☒ | The statistical test(s) used AND whether they are one- or two-sided<br>*Only common tests should be described solely by name; describe more complex techniques in the Methods section.* |
| ☒ | ☐ | A description of all covariates tested |
| ☐ | ☒ | A description of any assumptions or corrections, such as tests of normality and adjustment for multiple comparisons |
| ☐ | ☒ | A full description of the statistical parameters including central tendency (e.g. means) or other basic estimates (e.g. regression coefficient) AND variation (e.g. standard deviation) or associated estimates of uncertainty (e.g. confidence intervals) |
| ☐ | ☒ | For null hypothesis testing, the test statistic (e.g. *F*, *t*, *r*) with confidence intervals, effect sizes, degrees of freedom and *P* value noted<br>*Give P values as exact values whenever suitable.* |
| ☒ | ☐ | For Bayesian analysis, information on the choice of priors and Markov chain Monte Carlo settings |
| ☒ | ☐ | For hierarchical and complex designs, identification of the appropriate level for tests and full reporting of outcomes |
| ☒ | ☐ | Estimates of effect sizes (e.g. Cohen's *d*, Pearson's *r*), indicating how they were calculated |

*Our web collection on statistics for biologists contains articles on many of the points above.*

## Software and code

Policy information about availability of computer code

| | |
|---|---|
| Data collection | Light cycler R480; V1.5.0<br>Vilber -FUSION X acquisition software<br>RaptorX (http://raptorx.uchicago.edu/)<br>PSIPRED server (http://bioinf.cs.ucl.ac.uk/psipred/)<br>BD FACSDiva V9.0<br>BD Accuri C6 Software: V100.264.21<br>Cellsens Dimension Imaging software<br>Tecan i-control V1.10 |
| Data analysis | Light cycler R 480: V1.5.0<br>ImageJ-NIH.gov V1.53f51<br>Microsoft Excel 2019<br>GraphPad Prism 6<br>FLowJo V10.7.1<br>BDcFlow Plus<br>Data Visualization: Integrative Genomics Viewer from th Broad Institute IGV 2.8.12, PyMol 0.99rc6<br>DNASTAR Navigator 17.1.1.120-Clustal Omega<br>PSIPRED server (http://bioinf.cs.ucl.ac.uk/psipred/)<br>Big-FISH package (https://fish-quant.github.io/) |

For manuscripts utilizing custom algorithms or software that are central to the research but not yet described in published literature, software must be made available to editors and reviewers. We strongly encourage code deposition in a community repository (e.g. GitHub). See the Nature Portfolio guidelines for submitting code & software for further information.

## Data

Policy information about availability of data

All manuscripts must include a data availability statement. This statement should provide the following information, where applicable:

- Accession codes, unique identifiers, or web links for publicly available datasets
- A description of any restrictions on data availability
- For clinical datasets or third party data, please ensure that the statement adheres to our policy

RNA sequencing transcriptome data are available at GEO under accession code: GSE184399; Source data are provided with this paper

# Field-specific reporting

Please select the one below that is the best fit for your research. If you are not sure, read the appropriate sections before making your selection.

[x] Life sciences [ ] Behavioural & social sciences [ ] Ecological, evolutionary & environmental sciences

For a reference copy of the document with all sections, see nature.com/documents/nr-reporting-summary-flat.pdf

# Life sciences study design

All studies must disclose on these points even when the disclosure is negative.

| | |
|---|---|
| Sample size | Sample size of n≥3 biological replicates was chosen as repeats gave similar results and to increase power in statistical tests. Then, we have used at least three biological replicates for each experiment - unless stated otherwise as in Fig S3d when no effect was observed, or as in Fig 4b right when difference was observed in comparison to null. |
| Data exclusions | No data were excluded from the analysis |
| Replication | Experiments were independently replicated multiple times with reproducible results indicated in the figure legends. All attempts at replication were successful with appropriate positive and negative controls. |
| Randomization | Allocation to groups was random. |
| Blinding | Investigators were blinded to group allocation during data collection and analysis |

# Reporting for specific materials, systems and methods

We require information from authors about some types of materials, experimental systems and methods used in many studies. Here, indicate whether each material, system or method listed is relevant to your study. If you are not sure if a list item applies to your research, read the appropriate section before selecting a response.

## Materials & experimental systems

| n/a | Involved in the study | |
|---|---|---|
| [ ] | [x] | Antibodies |
| [ ] | [x] | Eukaryotic cell lines |
| [x] | [ ] | Palaeontology and archaeology |
| [x] | [ ] | Animals and other organisms |
| [x] | [ ] | Human research participants |
| [x] | [ ] | Clinical data |
| [x] | [ ] | Dual use research of concern |

## Methods

| n/a | Involved in the study | |
|---|---|---|
| [x] | [ ] | ChIP-seq |
| [ ] | [x] | Flow cytometry |
| [x] | [ ] | MRI-based neuroimaging |

## Antibodies

| | |
|---|---|
| Antibodies used | anti-Flag M2 (F1804-200UG, lot SLCD3990, Merck) 1/1000 ; anti-TASOR (HPA006735, lots A106822, C119001, Merck) 1/1000 – for IF assays: 1/500; anti-TASOR (HPA017142- Merck) for Immunoprecipitation experiments, anti-MPP8 (HPA040035, lot R38302, Merck) 1/500; Anticorps BrdU (IIB5) (For Nuclear Run On experiments: sc-32323, SantaCruz Biotechnology); anti-CNOT1 (For WB: 66507-1-Ig, Proteintech, 1/1000; For IP: 14276-1-AP, Proteintech); anti-CNOT7 (14102-1-AP, Proteintech) 1/500; anti-CNOT9 (22503-1-AP, Proteintech) 1/500; anti-DHX9 (17721-1-AP, Proteintech) 1/1000; anti-EXOSC10 (11178-1-AP, Proteintech) 1/1000; anti-HLTF (ab17984, Abcam) 1/1000; anti-IGF2BP1 (22803-1-AP, Proteintech) 1/1000; anti-KPNB1 (HPA029878-100ul, lot D114771, Merck) 1/1000; anti-MATR3 (12202-2-AP, Proteintech) 1/1000; anti-MORC2 (PA5-51172, TermoFisher) 1/1000; anti-Mouse IgG (for Nuclear Run On experiments: 12-371, Merck) anti-MTR4 (For WB and IP: 12719-2-AP, Proteintech) 1/1000; anti-PPHLN-1 (HPA038902, Lot A104626, Merck), anti-Rabbit IgG for nascent RNA IP (12-370, Merck) 1/1000; anti-RNAPII (For IP and WB: F12, sc-55492, Santa Cruz Biotechnology) 1/1000; anti-Ser2P-RNAPII (13499S, Cell Signaling technology) 1/1000 in 2.5% BSA-TBS-Tween 0.1%; anti-Ser5P- |

RNAPII (13523S, Cell Signaling technology) 1/1000 in 2.5% BSA-TBS-Tween 0.1%; anti-SUPT6H (For WB and IP: 23073-1-AP, Proteintech) 1/1000; anti-U1snRNP70 (sc-390899 (C3), Santa Cruz Biotechnology) 1/500; anti-YTHDF2 (24744-1-AP, Proteintech) 1/1000; anti-ZFC3H1 (HPA007151, Merck) 1/1000, anti-β-Actin (AC40, A3853, Merck) 1/1000; anti-αTubulin (T9026-.2mL, lot 081M4861, Merck) 1/1000; anti-GAPDH (6C5, SC-32233, Santa Cruz) 1/1000. All secondary antibodies anti-mouse (31430, lot VF297958, ThermoFisher) and anti-rabbit (31460, lots VC297287, UK293475 ThermoFisher) were used at a 1/10000 concentration

| | |
|---|---|
| Validation | all primary antibodies have been used in techniques for which they have been validated by the manufacturer or supplier except for the anti-Human TASOR (HPA017142- Merck) which we have validated for endogenous immunoprecipitation (Figs 2c and 3a), and the anti-Human TASOR (HPA006735) which we have valided for Immunofluorescence use (Figs 7a, S4c). |

# Eukaryotic cell lines

Policy information about cell lines

| | |
|---|---|
| Cell line source(s) | HeLa HIV-1 LTRΔTAR-Luc cells were generated in the laboratory of Stéphane Emiliani from the HeLa HIV-1 LTR-Luc cells described by du Chené et al., 2007; HeLa (CCL-2), HEK293T (CRL-3216), THP-1 (TIB-202) and Jurkat E6 clone (TIB-152) cells were purchased from ATCC |
| Authentication | Cells were checked permanently according to morphology and functional features though they were not authenticated in the laboratory. Luciferase expression from the HeLa HIV-1 LTRΔTAR-Luc, or HIV-1 LTR-Luc cells was regularly checked |
| Mycoplasma contamination | Cell lines are regularly test for mycoplasma contamination (MycoAlert, Lonza and PCR). If contaminated, cells are discarded. Experiments were performed with non-contaminated cells. |
| Commonly misidentified lines (See ICLAC register) | No commonly misidentified cell lines were used |

# Flow Cytometry

## Plots

Confirm that:

- [x] The axis labels state the marker and fluorochrome used (e.g. CD4-FITC).
- [x] The axis scales are clearly visible. Include numbers along axes only for bottom left plot of group (a 'group' is an analysis of identical markers).
- [x] All plots are contour plots with outliers or pseudocolor plots.
- [x] A numerical value for number of cells or percentage (with statistics) is provided.

## Methodology

| | |
|---|---|
| Sample preparation | Cells were havested by centrifugation, washed in PBS and resuspended in PBS-EDTA 0.5mM |
| Instrument | ACCURI C6 (BD accuri) and BD LSR Fortessa |
| Software | BD FACSDiva V9.0<br>BD Accuri C6 Software: V100.264.21 |
| Cell population abundance | 10,000 events were collected within the P1 living cell population determined by SSC and FSC. For the J-Lat A1 and Jurkat HIV-1 EGFP, analyses were performed on the whole GFP+ population that ranges from 5% if untreated up to 90% upon treatment with TNF/ and depletion of CNOT1. |
| Gating strategy | FSC/SSC gating strategy depends on the cell type. For GFP-positive gating, negative controls were used: untreated J-Lat A1 cells; and uninfected Jurkat cells. The gating is arbitrary but the same gate is maintained for all condition |

- [x] Tick this box to confirm that a figure exemplifying the gating strategy is provided in the Supplementary Information.
