# Supplementary material for: TASOR epigenetic repressor cooperates with a CNOT1 RNA degradation pathway to repress HIV
Source: Nat Commun. 2022 Jan 10;13:66. doi: 10.1038/s41467-021-27650-5 (PMC8748822; doi:10.1038/s41467-021-27650-5)

Fig 1b

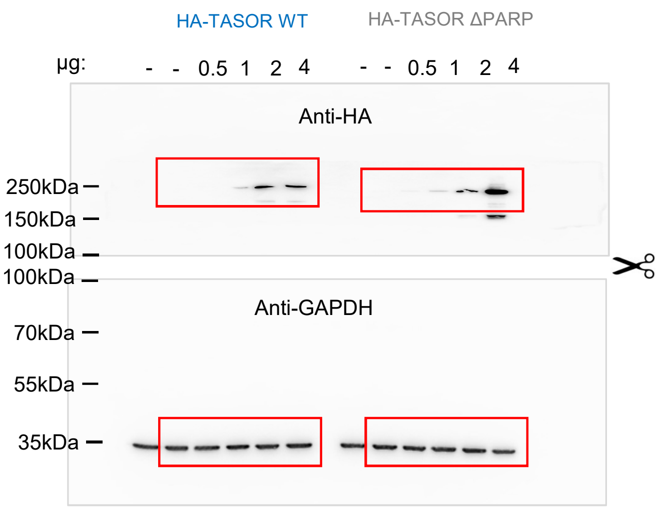

Fig 1c

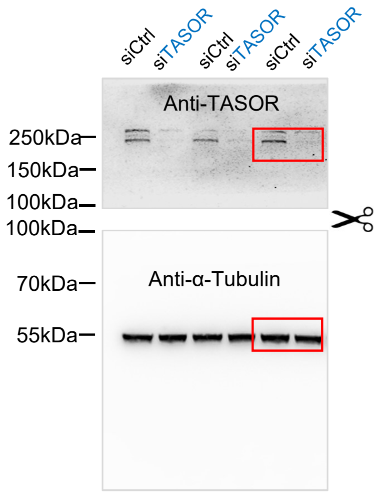

Fig 1d

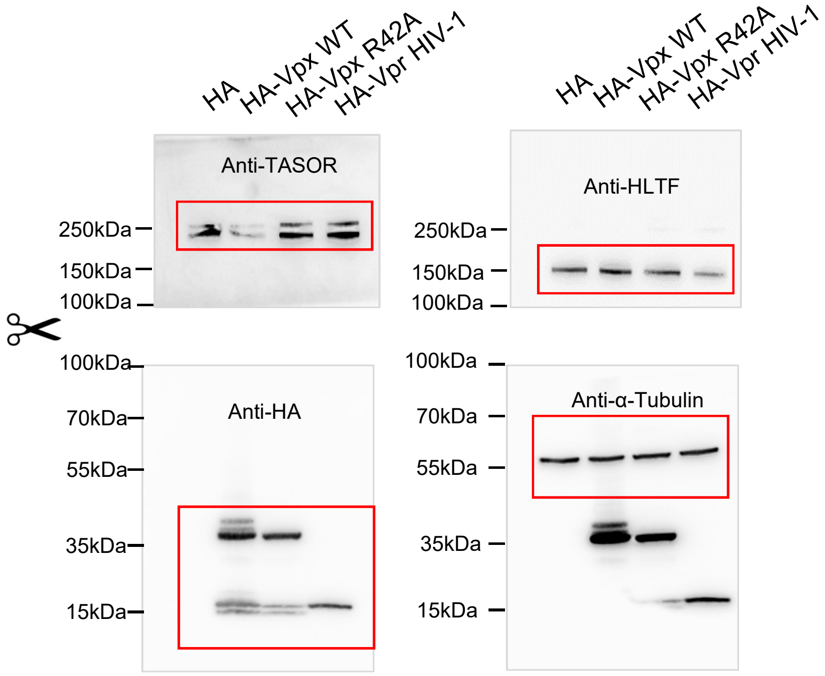

Fig 2b

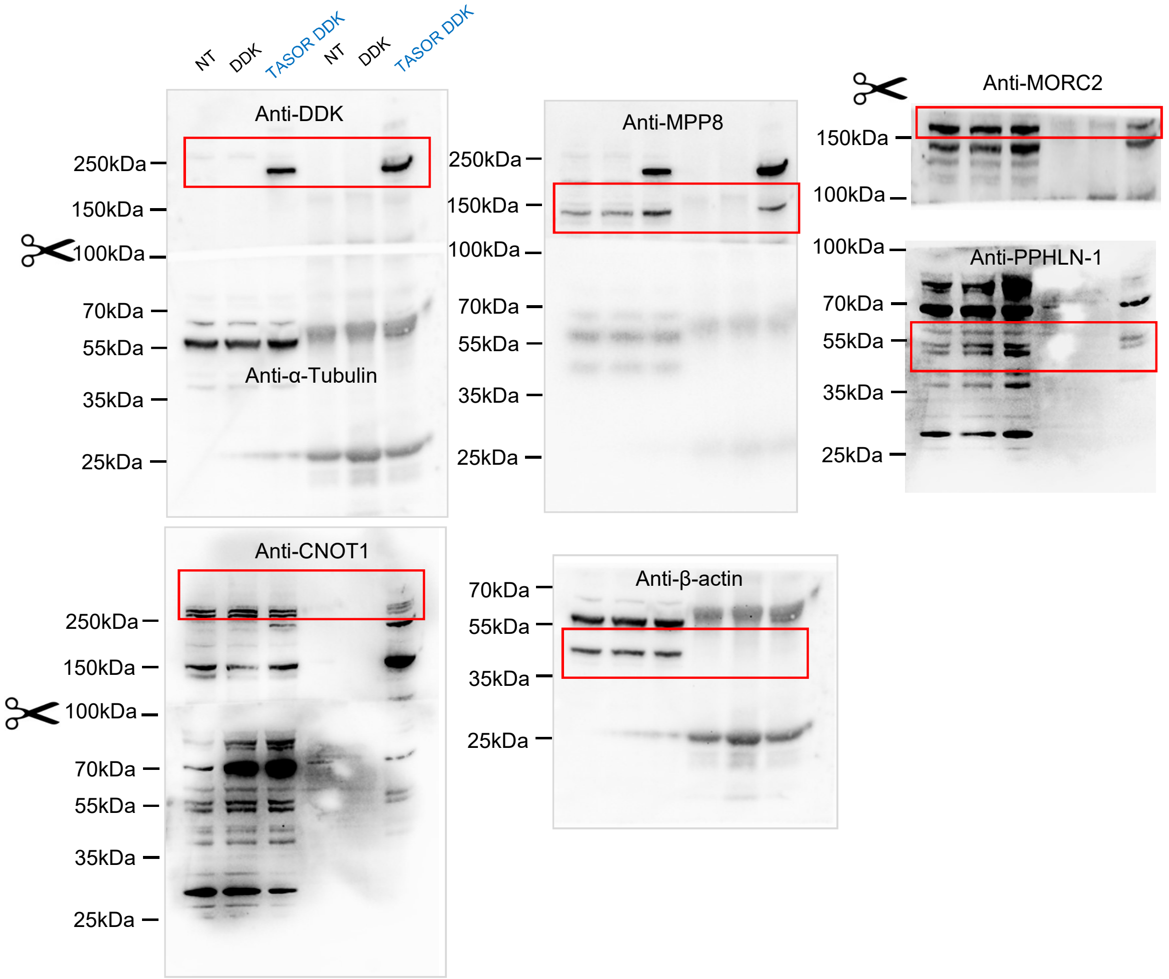

Fig 2c

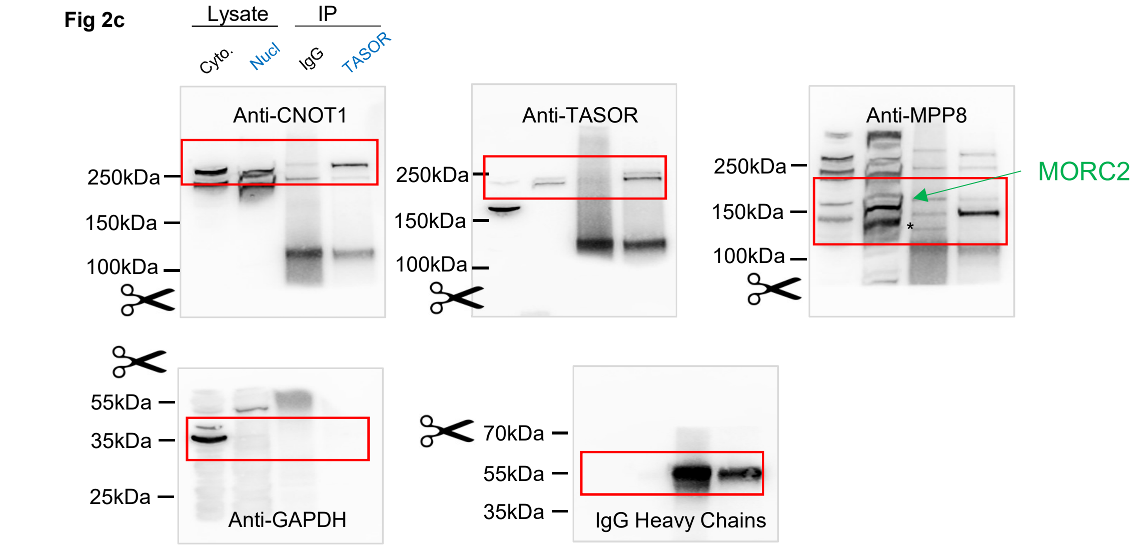

Fig 2d

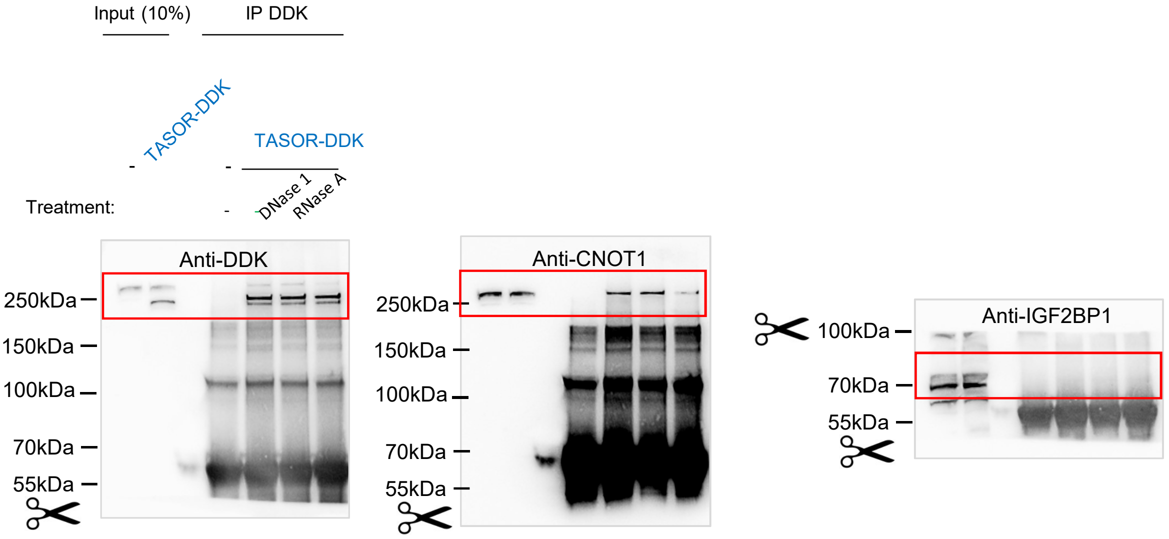

Fig 2e

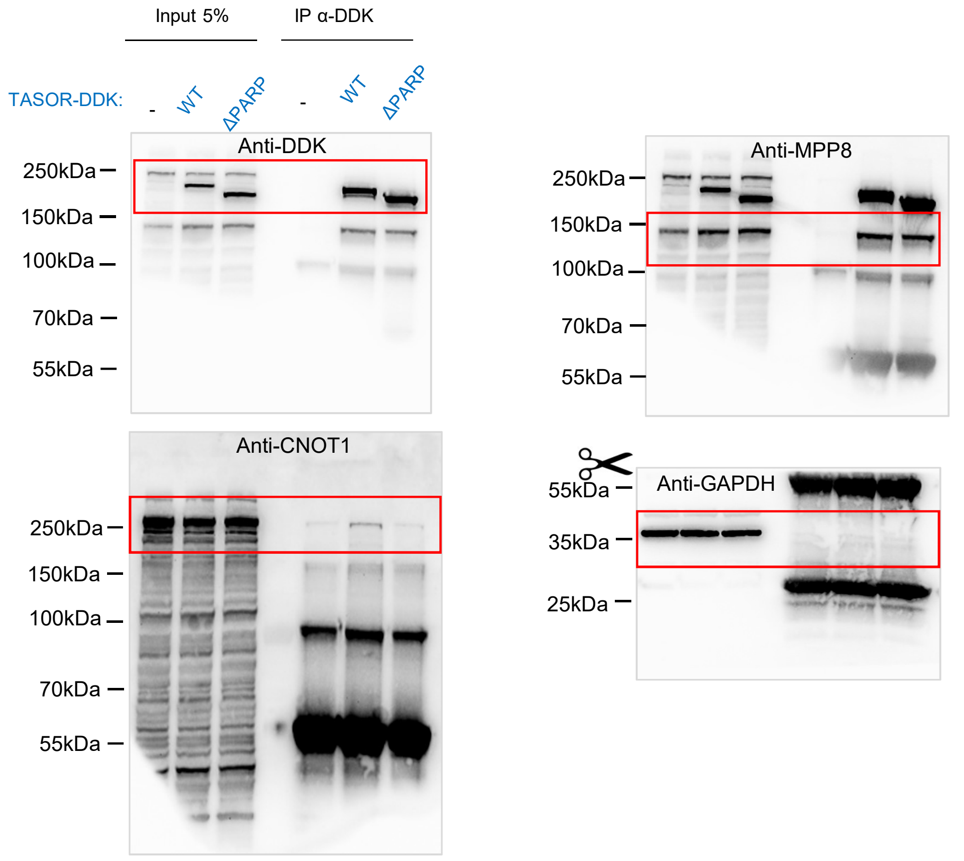

Fig 3a

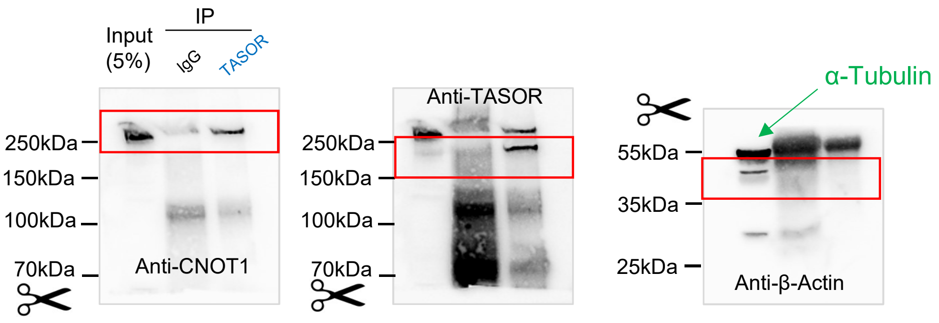

Fig 3d

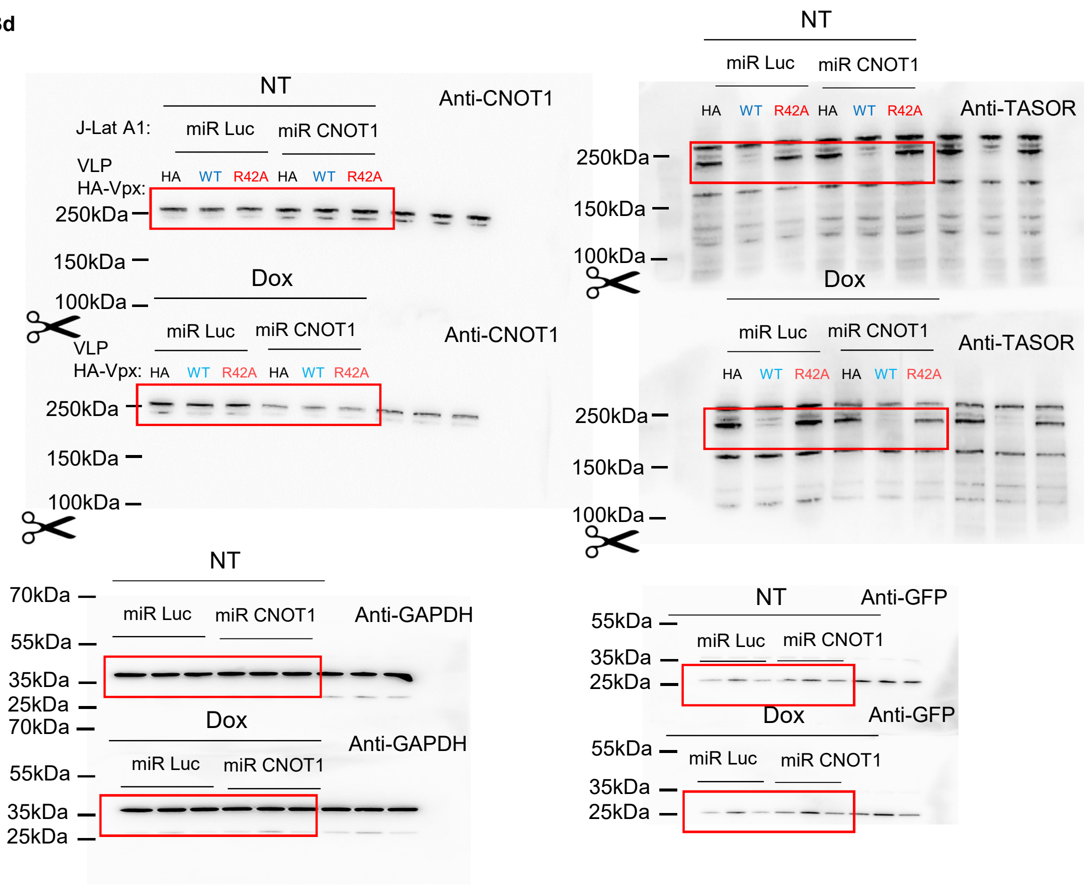

Fig 4c

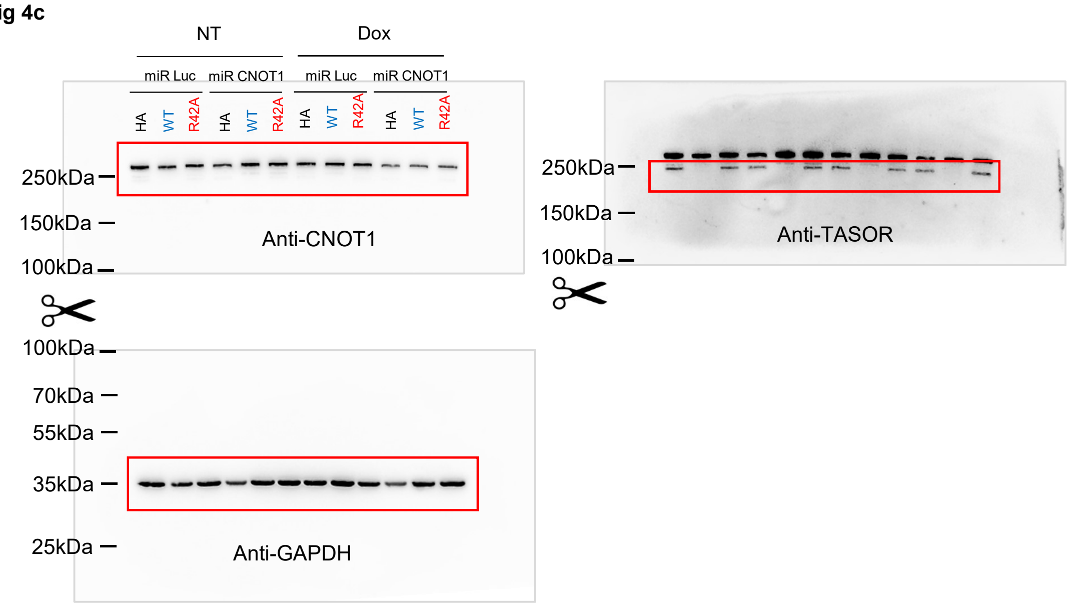
NT
Dox
miR Luc
miR CNOT1
miR Luc
miR CNOT1
HA
WT
R42A
HA
WT
R42A
HA
WT
R42A
HA
WT
R42A
250kDa
150kDa
100kDa
Anti-CNOT1
250kDa
150kDa
100kDa
Anti-TASOR
100kDa
70kDa
55kDa
35kDa
25kDa
Anti-GAPDH

Fig 5a

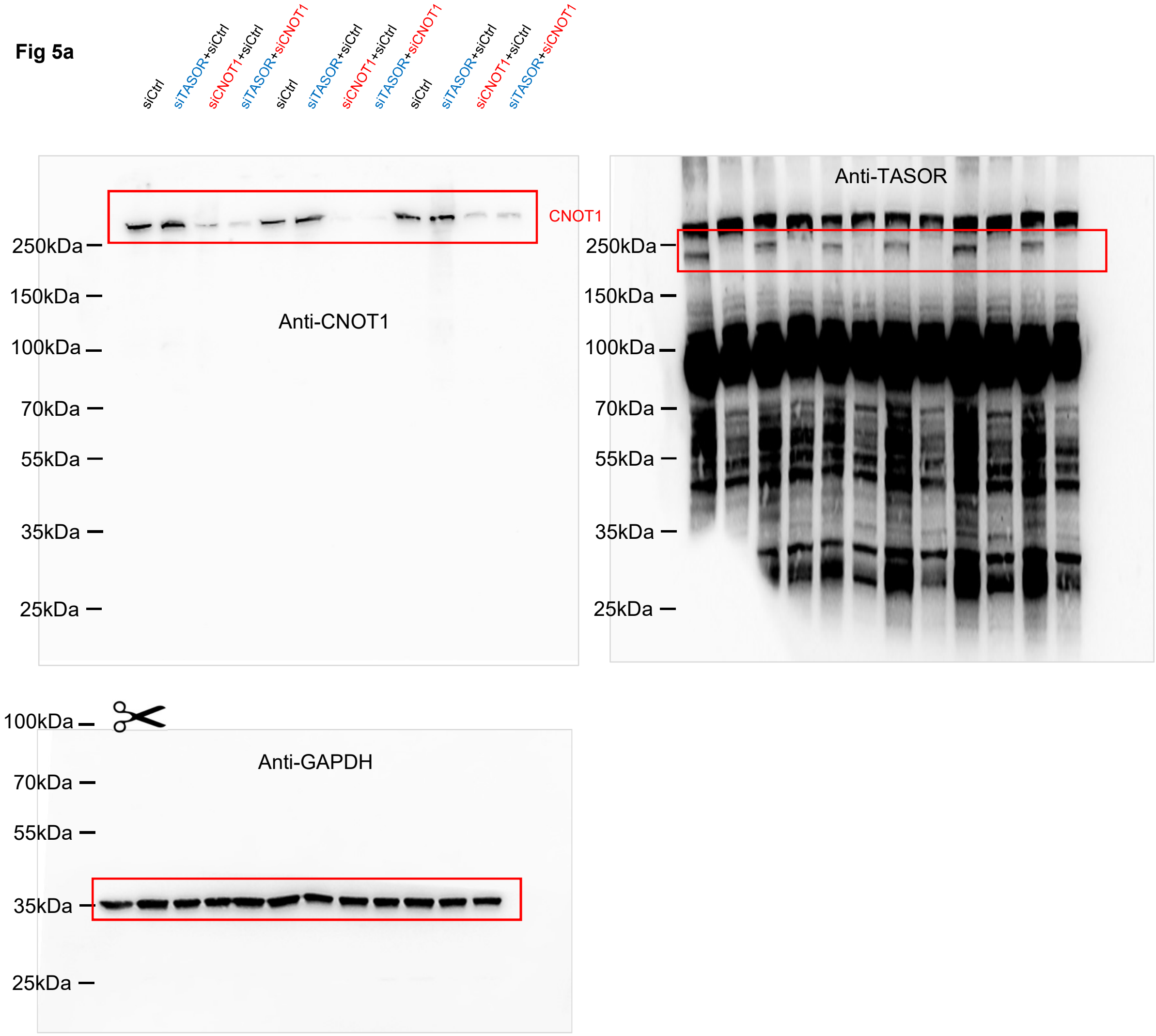
siCtrl
siTASOR+siCtrl
siCNOT1+siCtrl
siTASOR+siCNOT1
siCtrl
siTASOR+siCtrl
siCNOT1+siCtrl
siTASOR+siCNOT1
siCtrl
siTASOR+siCtrl
siCNOT1+siCtrl
siTASOR+siCNOT1
CNOT1
250kDa
150kDa
100kDa
70kDa
55kDa
35kDa
25kDa
Anti-CNOT1
Anti-TASOR
250kDa
150kDa
100kDa
70kDa
55kDa
35kDa
25kDa
100kDa
70kDa
55kDa
35kDa
25kDa
Anti-GAPDH

Fig 6a

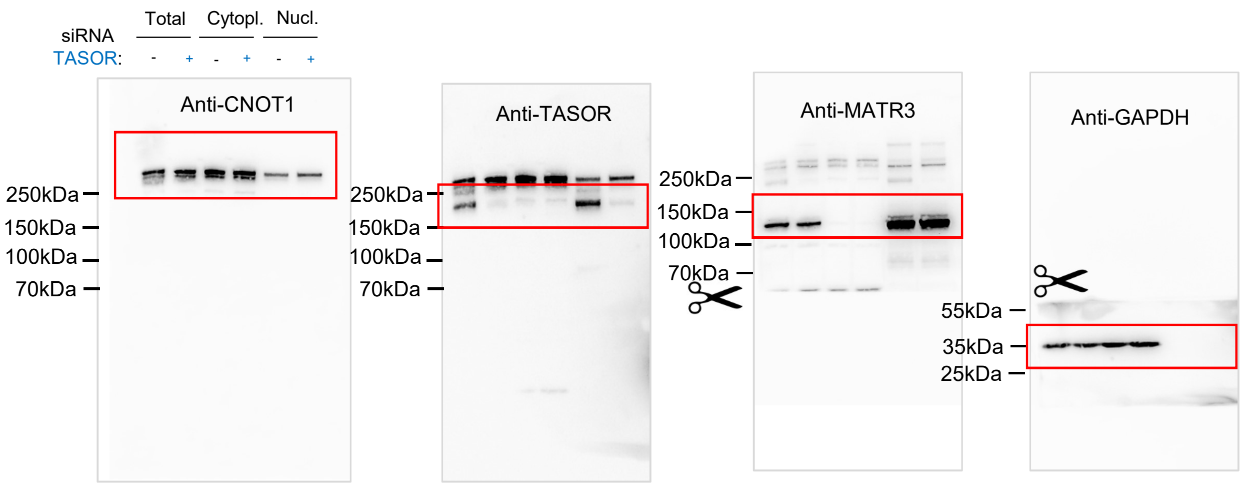

Fig 6b

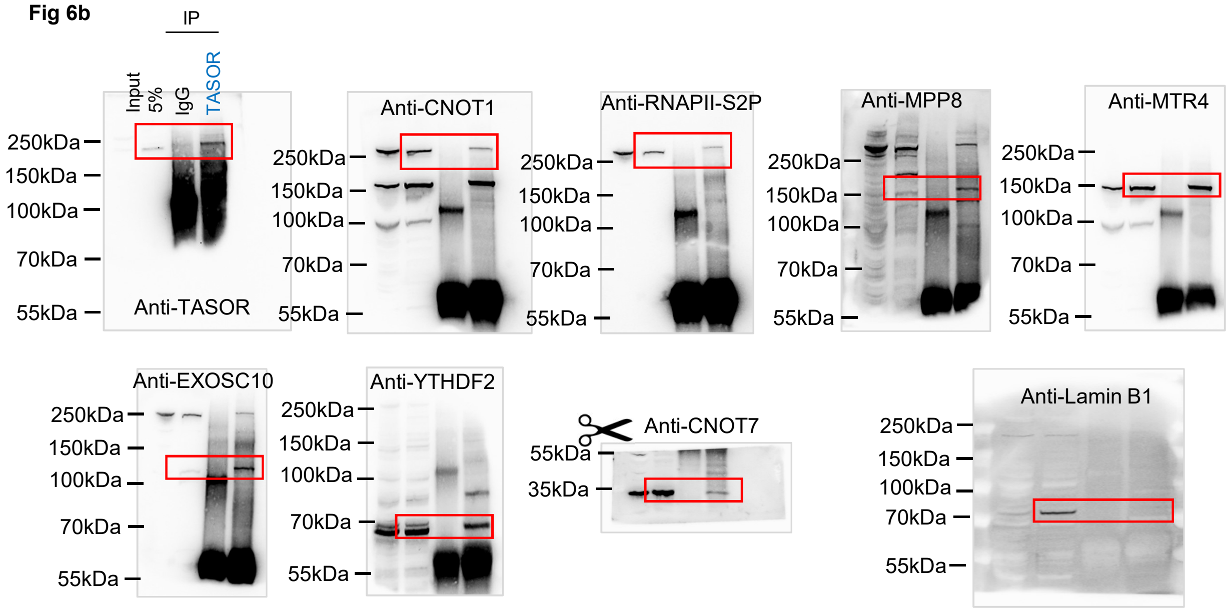

Fig 6c

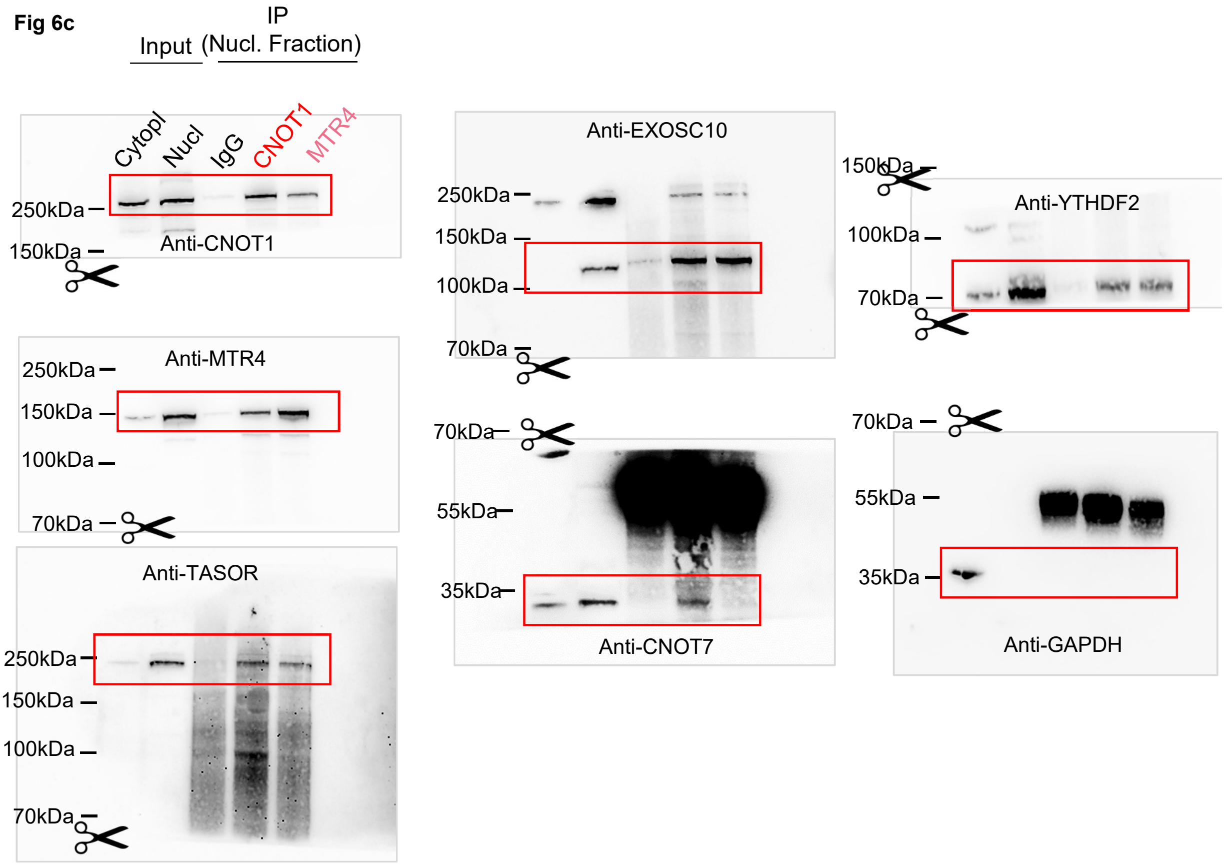

Fig 6d

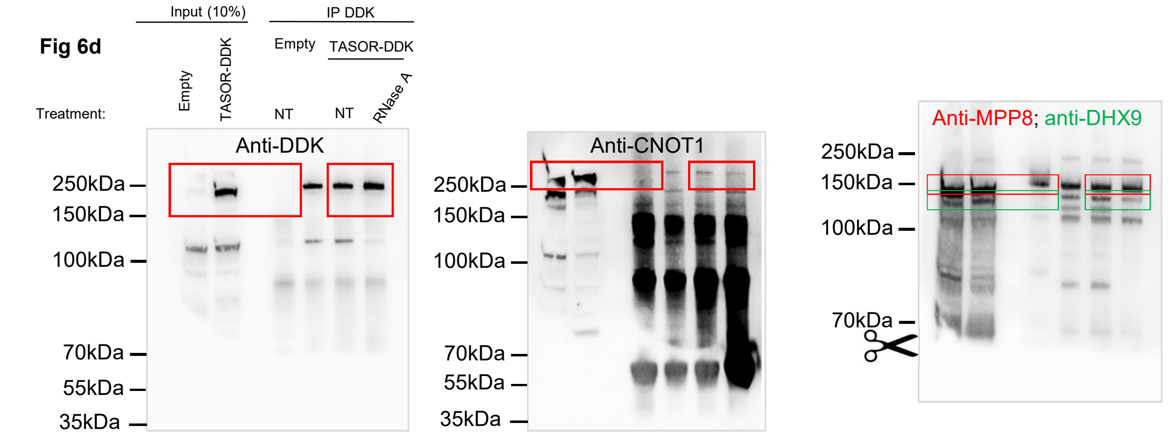
Input (10%)
IP DDK
Empty
TASOR-DDK
Empty
TASOR-DDK
Treatment:
NT
NT
RNase A
Anti-DDK
Anti-CNOT1
Anti-MPP8; anti-DHX9
250kDa
150kDa
100kDa
70kDa
55kDa
35kDa

Fig 6e

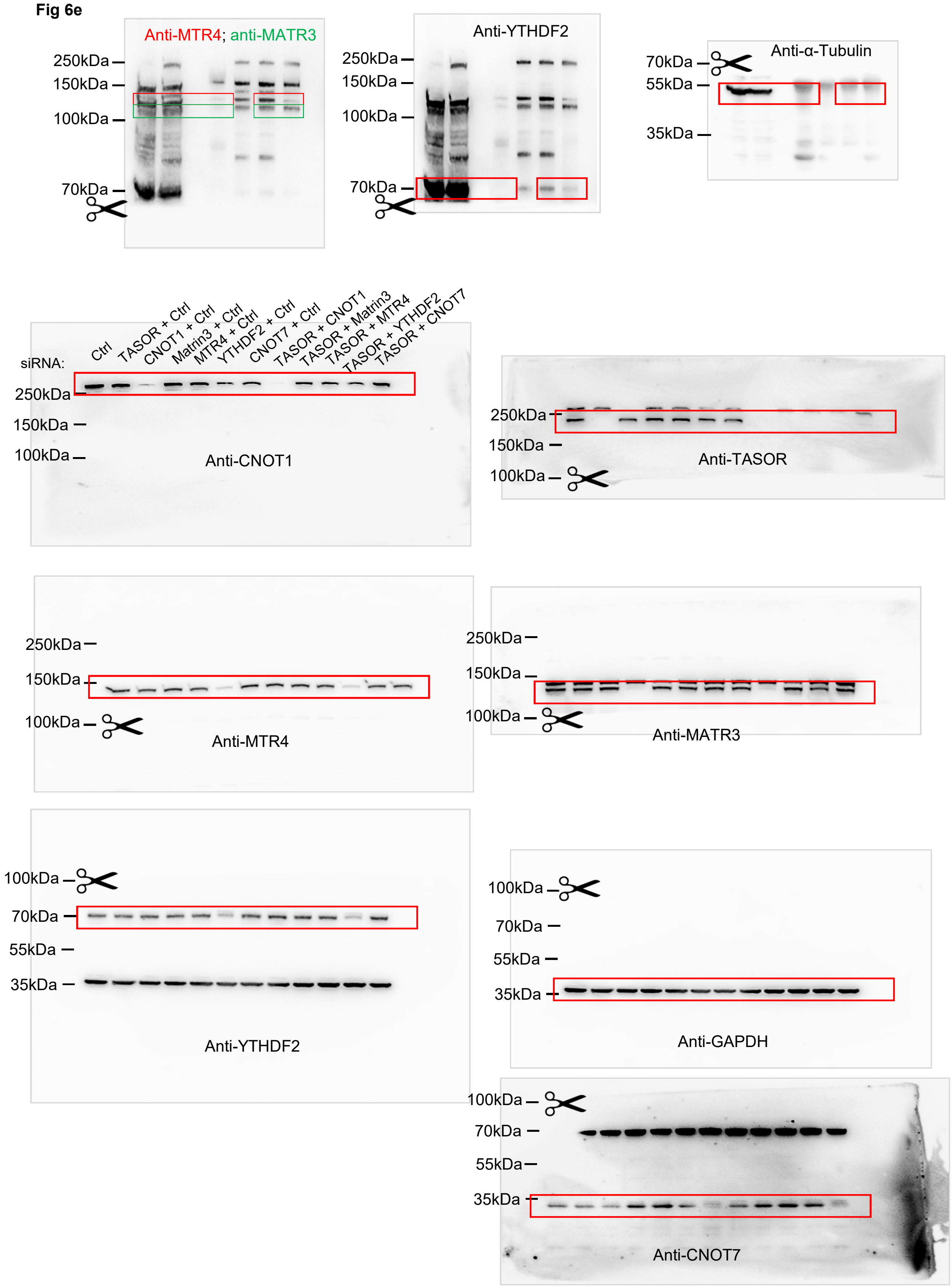
Anti-MTR4; anti-MATR3
Anti-YTHDF2
Anti-α-Tubulin
250kDa
150kDa
100kDa
70kDa
55kDa
35kDa
siRNA:
Ctrl
TASOR + Ctrl
CNOT1 + Ctrl
Matrin3 + Ctrl
MTR4 + Ctrl
YTHDF2 + Ctrl
CNOT7 + Ctrl
TASOR + CNOT1
TASOR + Matrin3
TASOR + MTR4
TASOR + YTHDF2
TASOR + CNOT7
Anti-CNOT1
Anti-TASOR
Anti-MTR4
Anti-MATR3
Anti-YTHDF2
Anti-GAPDH
Anti-CNOT7

Fig 7c

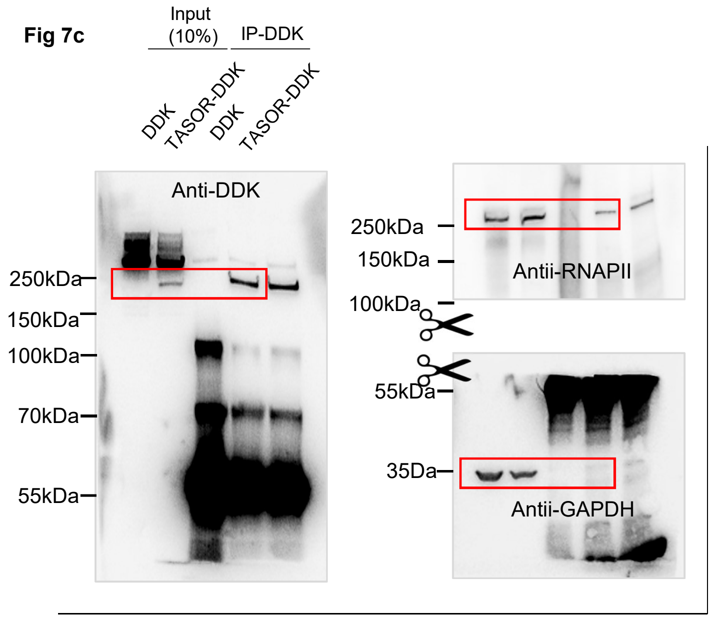

Fig 7d

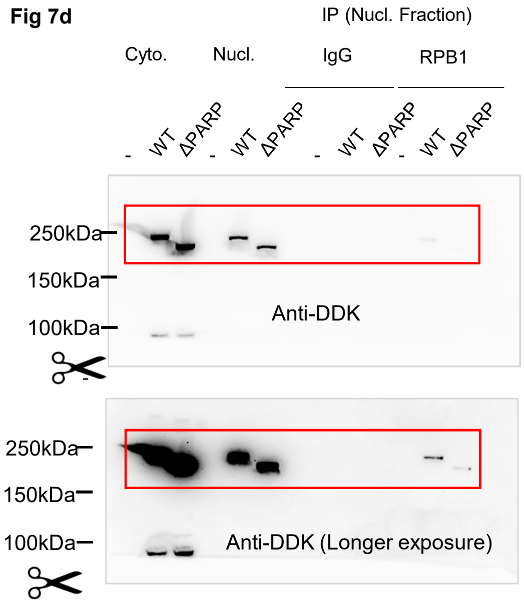

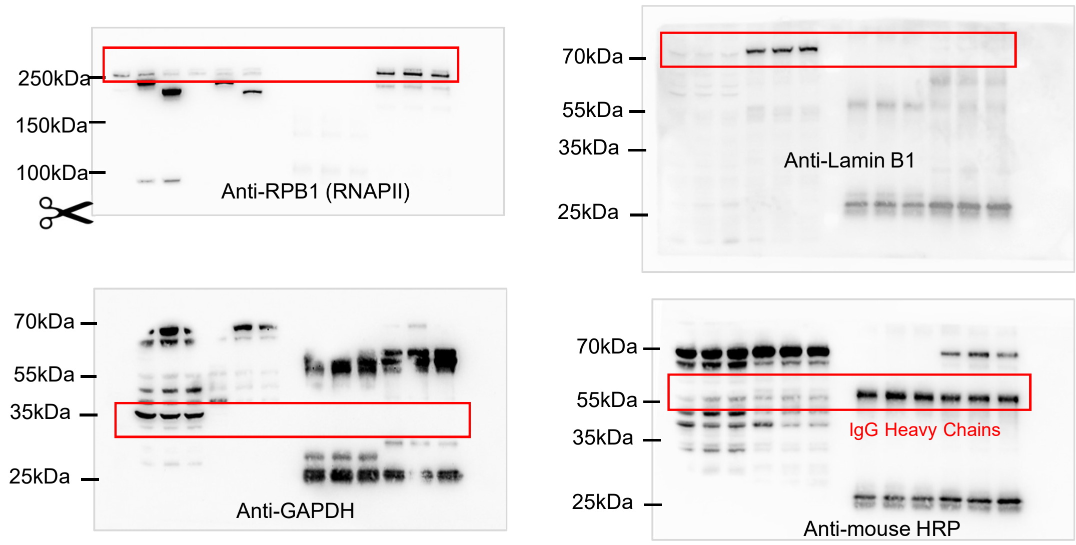

Fig 7e

HEK293T

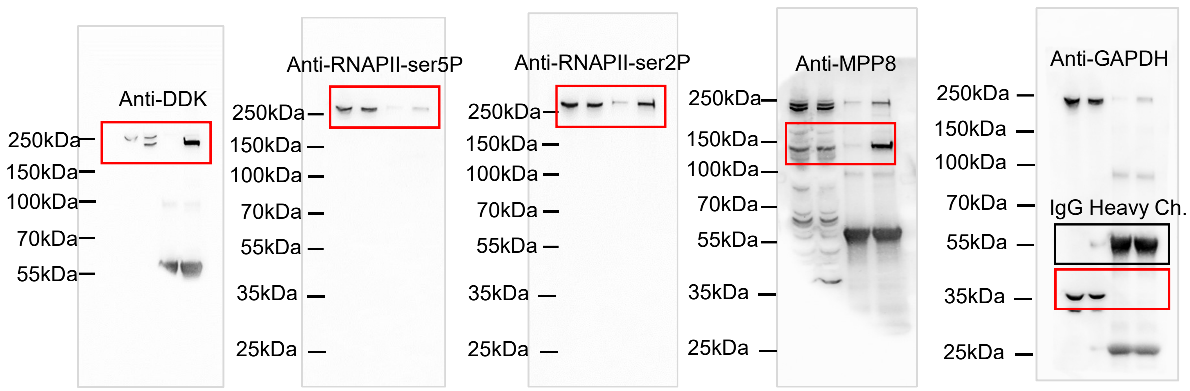

HeLa LTR-ΔTAR-Luc

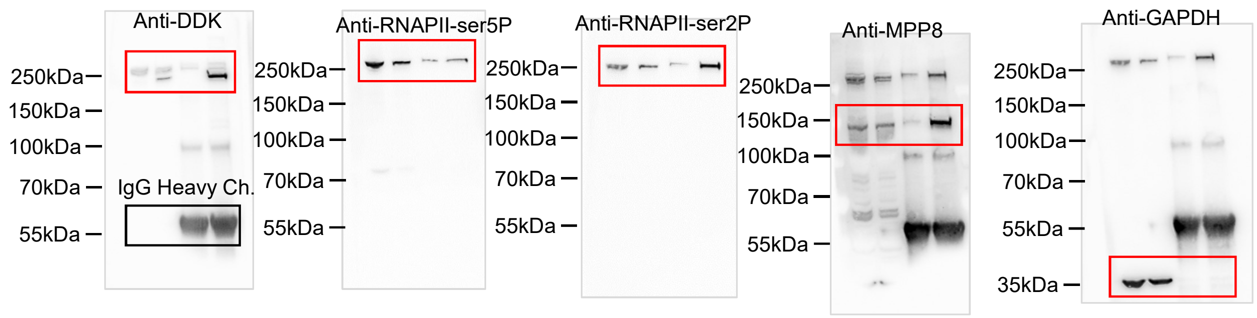

Fig 7f

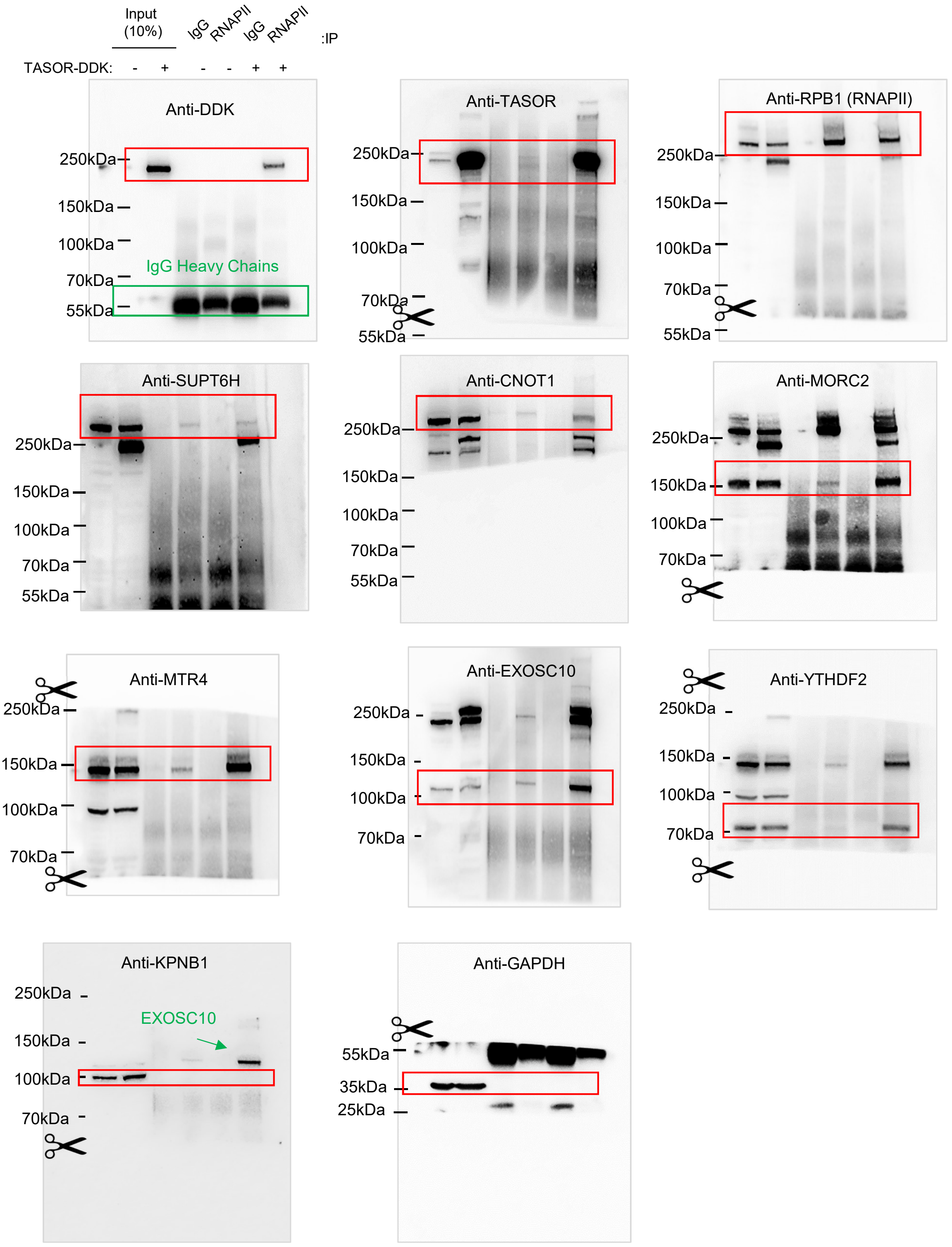
Input (10%)
IgG
RNAPII
IgG
RNAPII
:IP
TASOR-DDK: - + - - + +
Anti-DDK
250kDa
150kDa
100kDa
70kDa
55kDa
IgG Heavy Chains
Anti-TASOR
250kDa
150kDa
100kDa
70kDa
55kDa
Anti-RPB1 (RNAPII)
250kDa
150kDa
100kDa
70kDa
55kDa
Anti-SUPT6H
250kDa
150kDa
100kDa
70kDa
55kDa
Anti-CNOT1
250kDa
150kDa
100kDa
70kDa
55kDa
Anti-MORC2
250kDa
150kDa
100kDa
70kDa
Anti-MTR4
250kDa
150kDa
100kDa
70kDa
Anti-EXOSC10
250kDa
150kDa
100kDa
70kDa
Anti-YTHDF2
250kDa
150kDa
100kDa
70kDa
Anti-KPNB1
250kDa
150kDa
100kDa
70kDa
EXOSC10
Anti-GAPDH
55kDa
35kDa
25kDa

Fig 7g

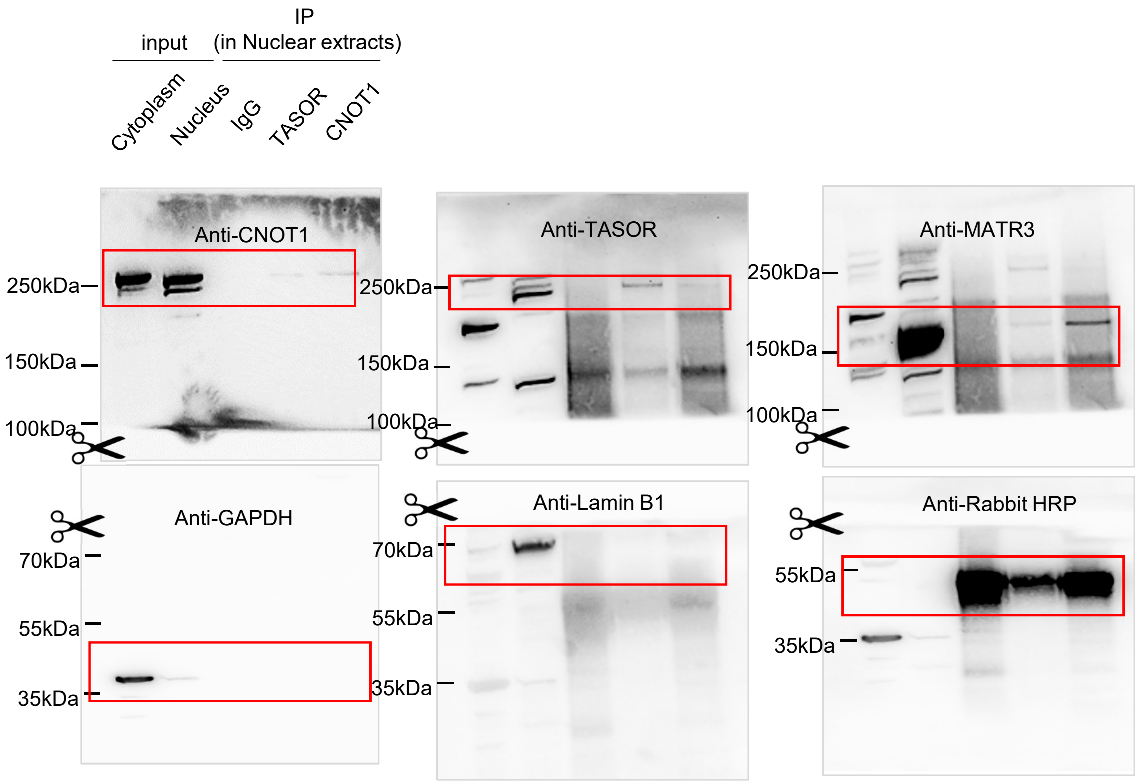
IP
input
(in Nuclear extracts)
Cytoplasm
Nucleus
IgG
TASOR
CNOT1
Anti-CNOT1
250kDa
150kDa
100kDa
Anti-TASOR
250kDa
150kDa
100kDa
Anti-MATR3
250kDa
150kDa
100kDa
Anti-GAPDH
70kDa
55kDa
35kDa
Anti-Lamin B1
70kDa
55kDa
35kDa
Anti-Rabbit HRP
55kDa
35kDa

Fig S1b

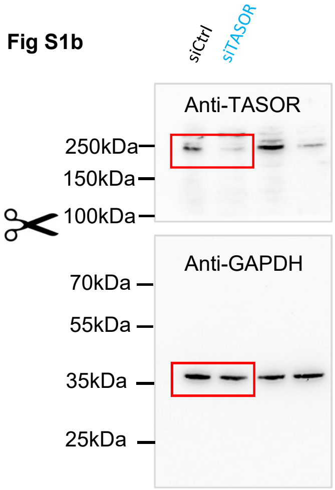

Fig S1e, left

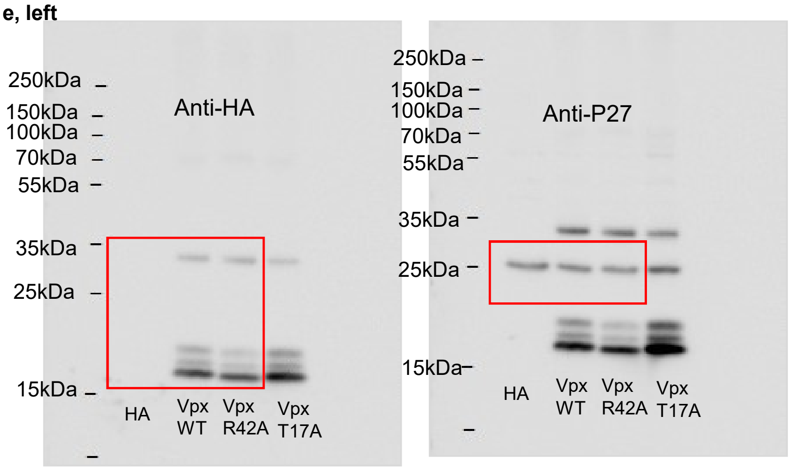

Fig S1e, right

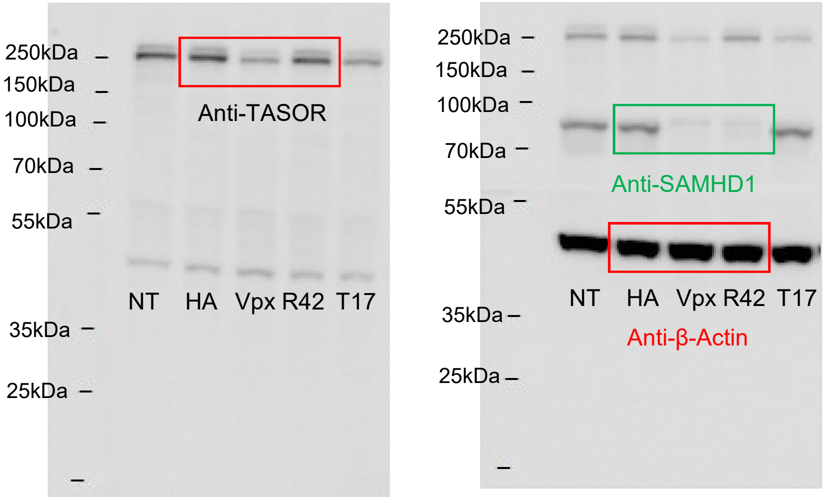

Fig S2d

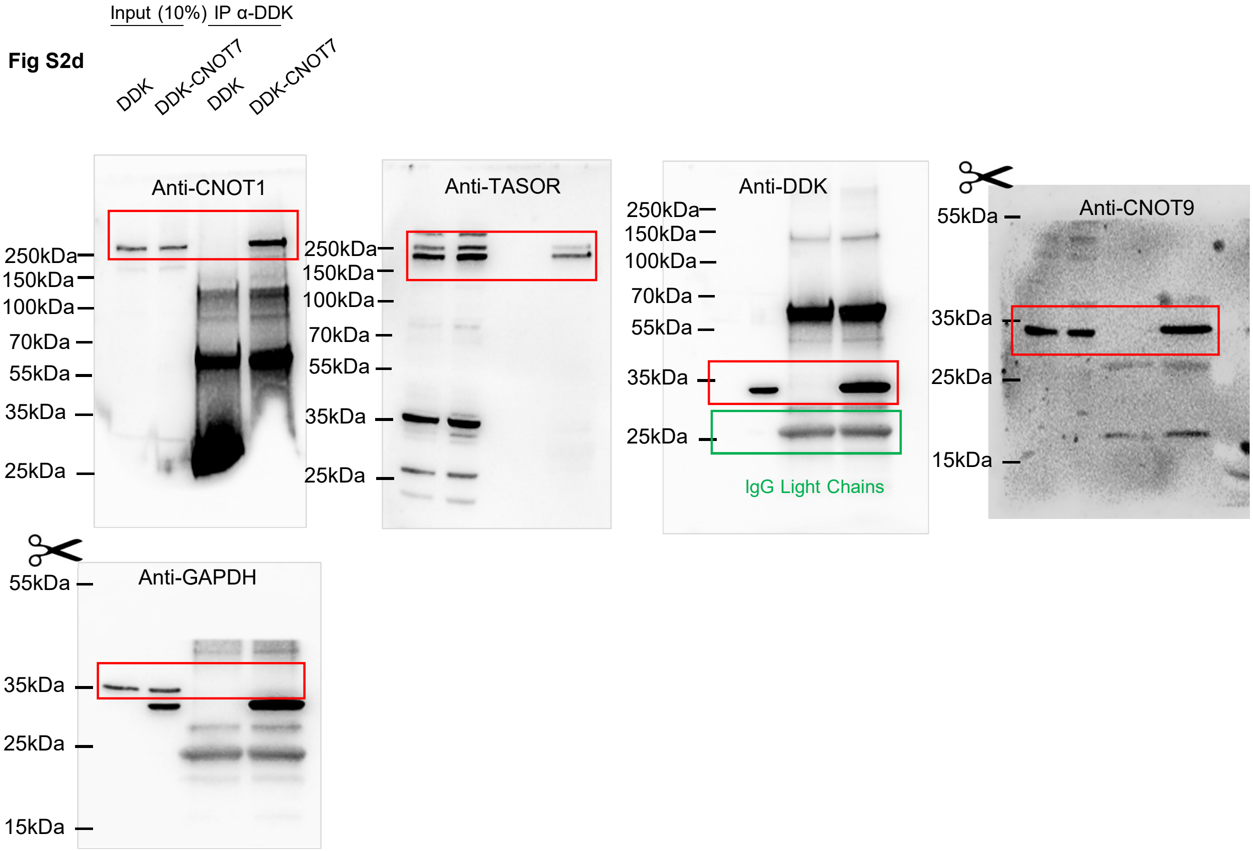

Fig S2e

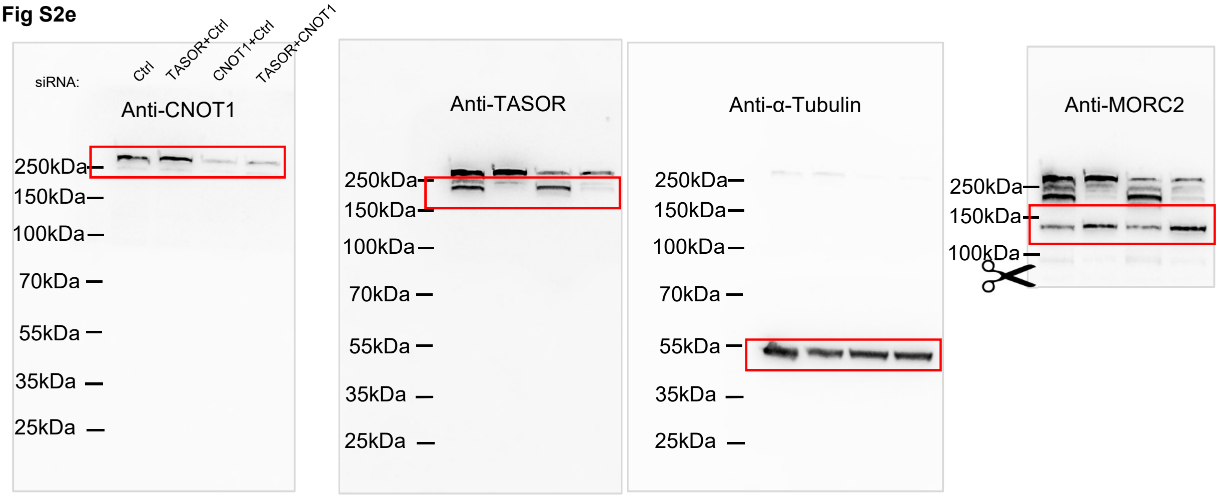

Fig S2f

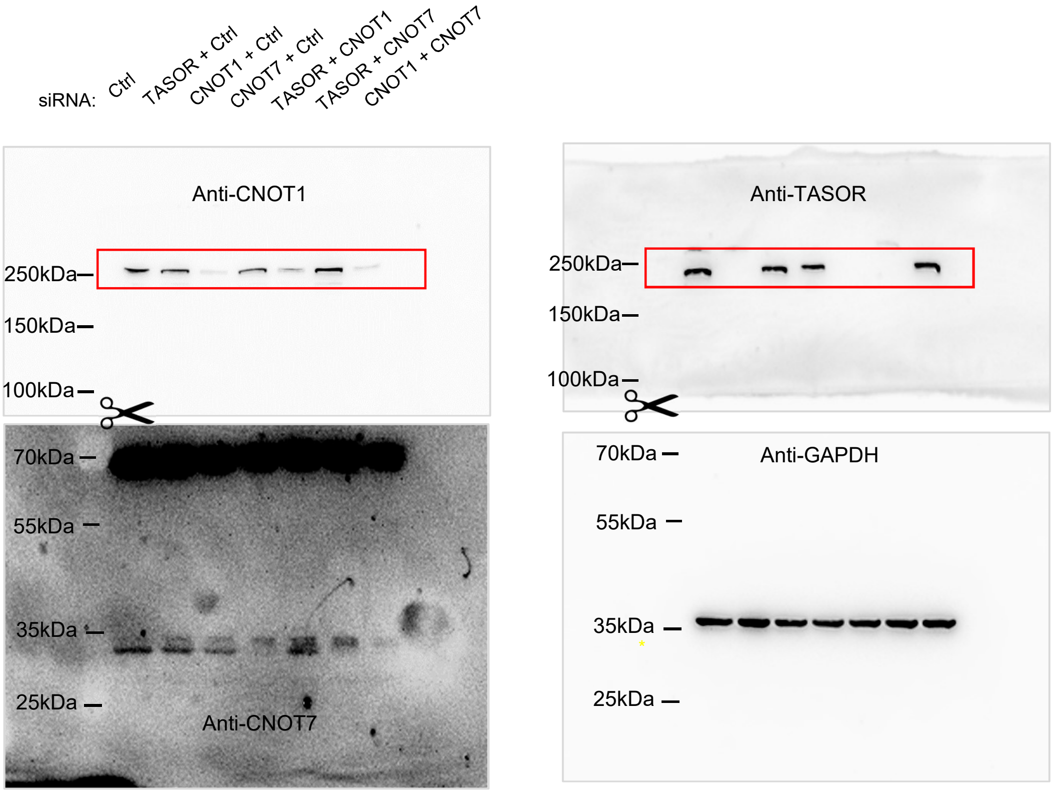

Fig S2g

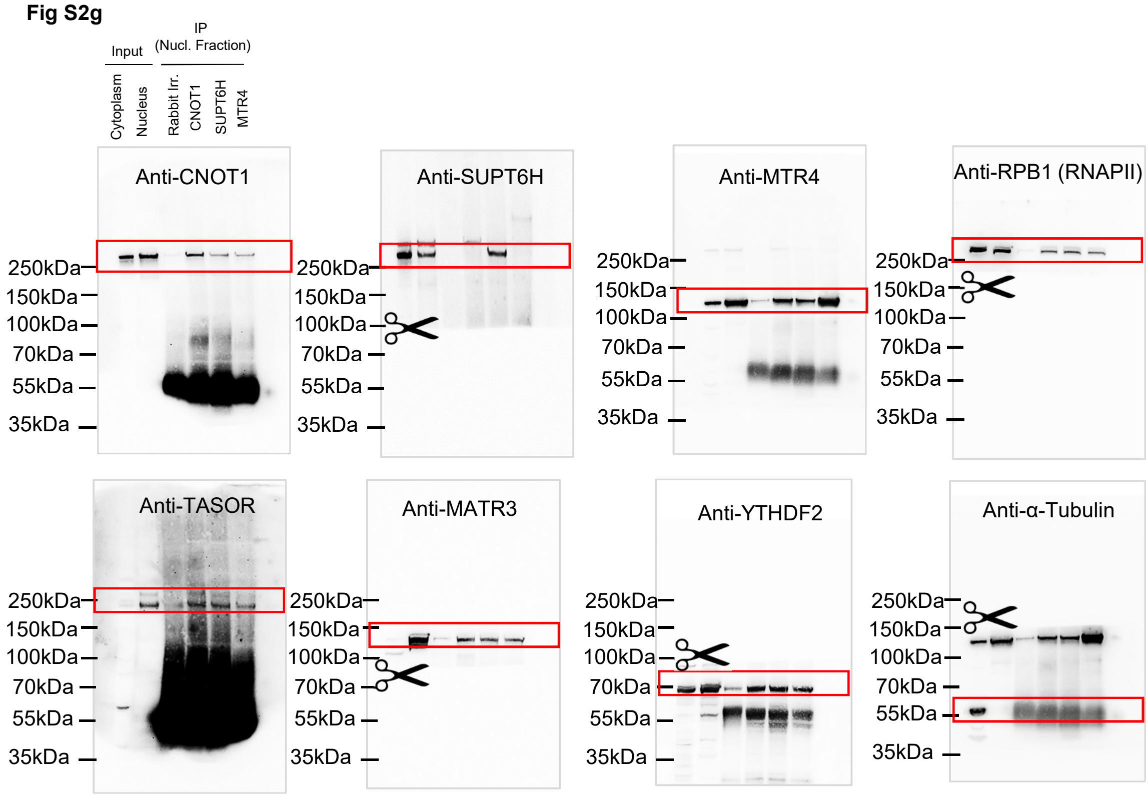
Input
IP (Nucl. Fraction)
Cytoplasm
Nucleus
Rabbit Irr.
CNOT1
SUPT6H
MTR4
Anti-CNOT1
Anti-SUPT6H
Anti-MTR4
Anti-RPB1 (RNAPII)
Anti-TASOR
Anti-MATR3
Anti-YTHDF2
Anti-α-Tubulin
250kDa
150kDa
100kDa
70kDa
55kDa
35kDa

Fig S3a

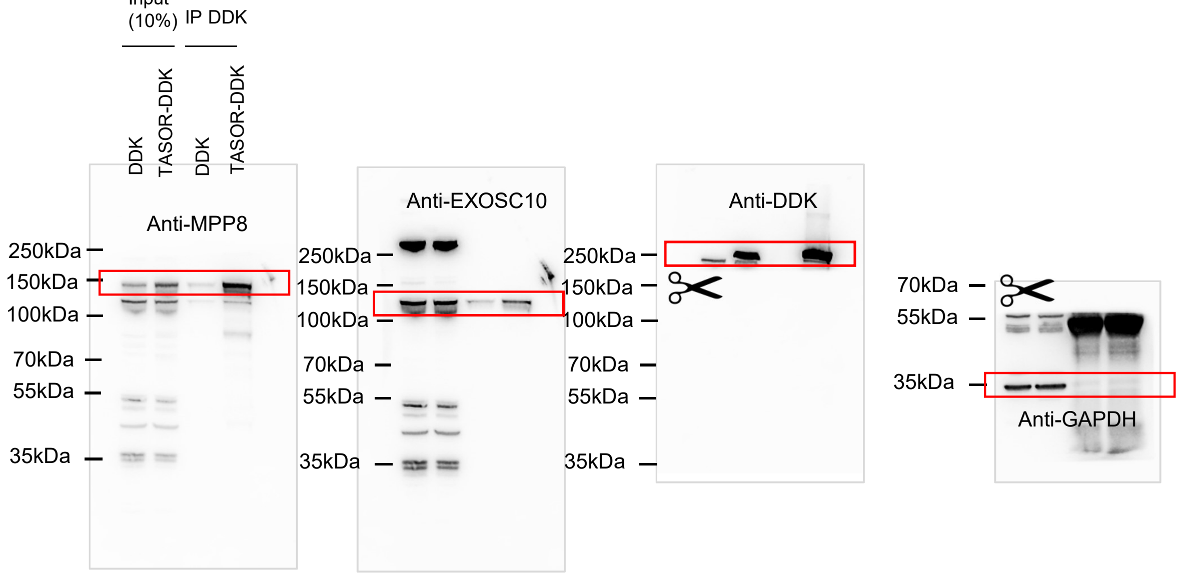

Fig S3b

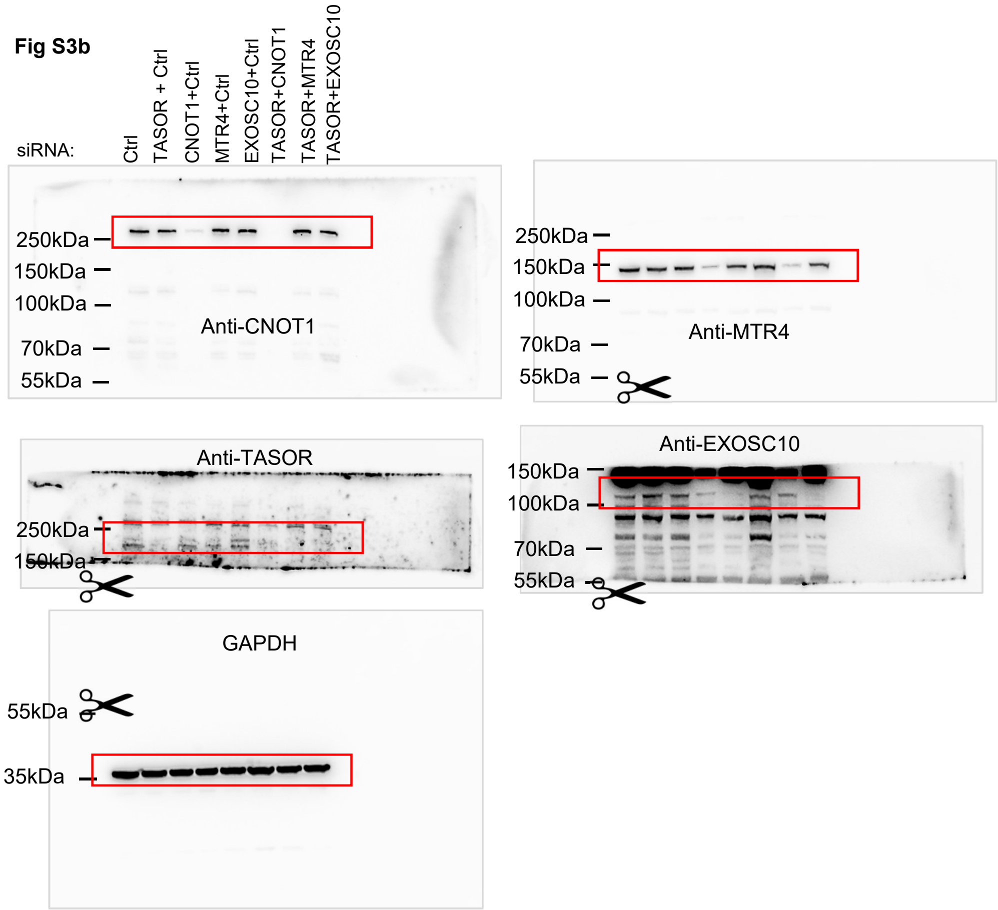

Fig S3c

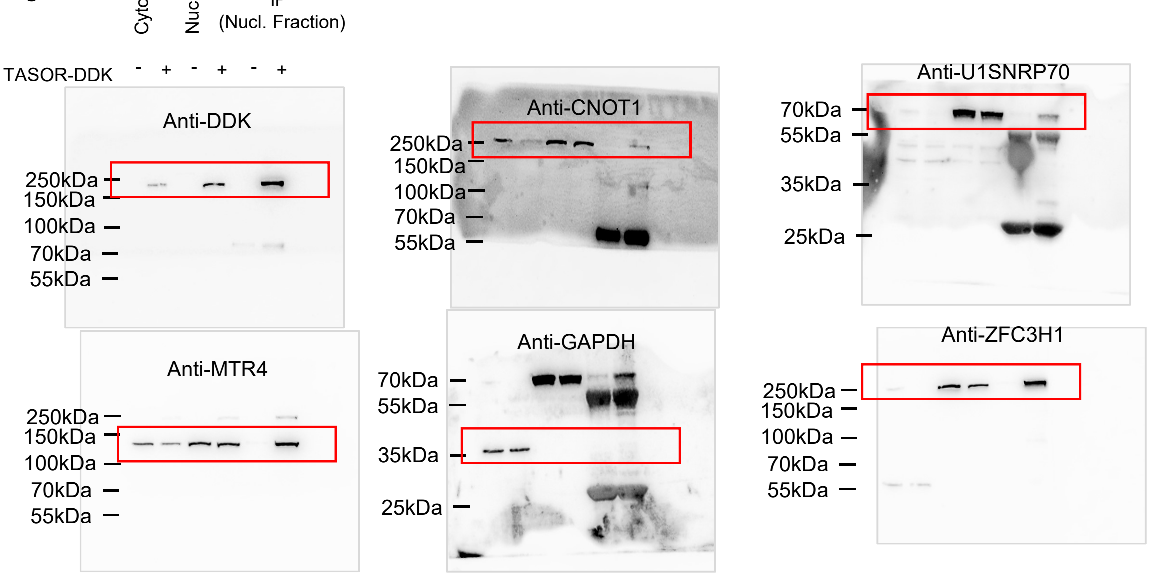

Fig S3d

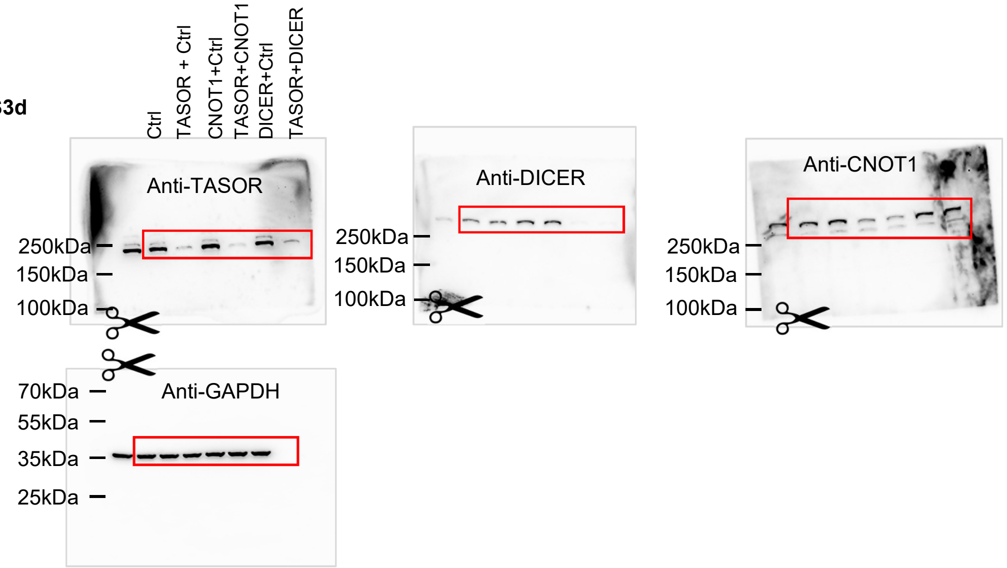

Fig S4a

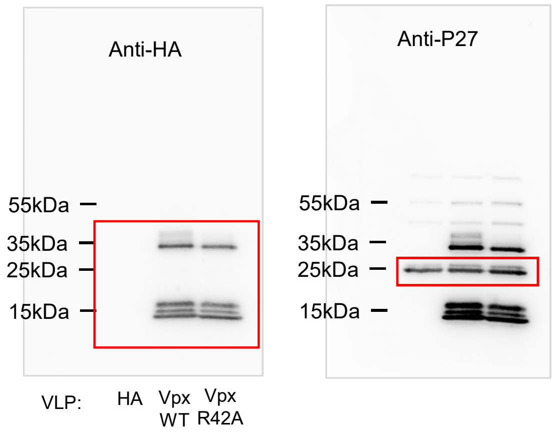

Fig S4b

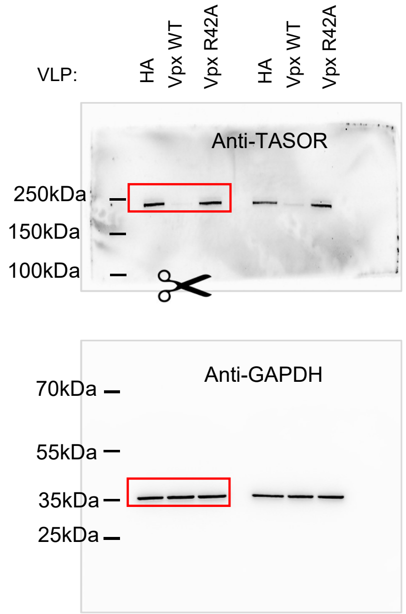

Fig S4h

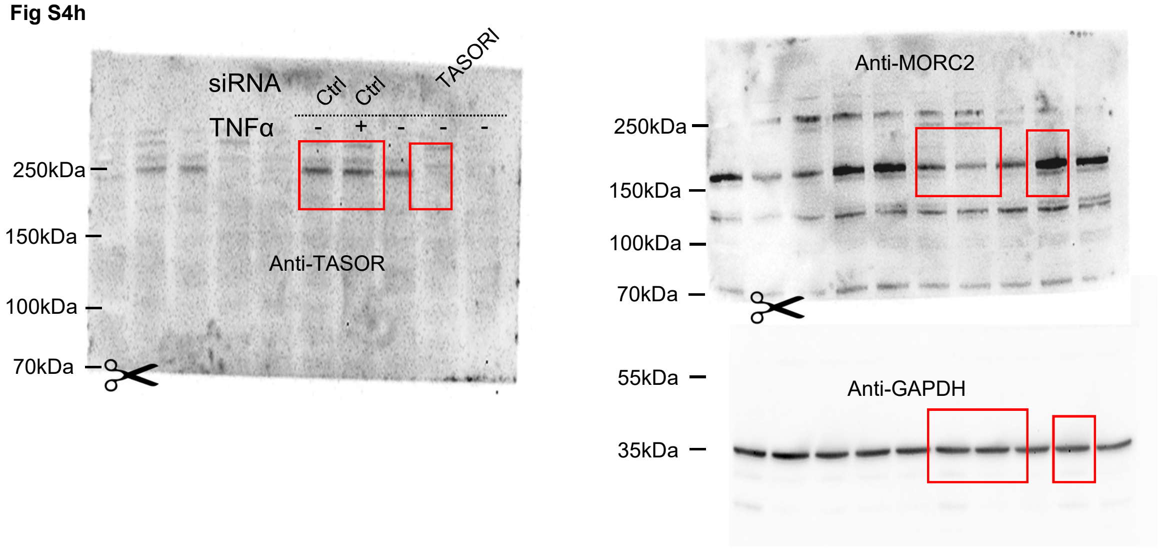

Supplement: Supplementary file 4 — Uncropped Western Blot Matkovic [file 41467_2021_27650_MOESM4_ESM.pdf]
